# Vaccine delivery alerts innate immune systems for more immunogenic vaccination

**DOI:** 10.1172/jci.insight.144627

**Published:** 2021-04-08

**Authors:** Zhuofan Li, Yan Cao, Yibo Li, Yiwen Zhao, Xinyuan Chen

**Affiliations:** Department of Biomedical and Pharmaceutical Sciences, College of Pharmacy, University of Rhode Island, Kingston, Rhode Island, USA.

**Keywords:** Vaccines, Adaptive immunity

## Abstract

Vaccine delivery technologies are mainly designed to minimally invasively deliver vaccines to target tissues with little or no adjuvant effects. This study presents a prototype laser-based powder delivery (LPD) with inherent adjuvant effects for more immunogenic vaccination without incorporation of external adjuvants. LPD takes advantage of aesthetic ablative fractional laser to generate skin microchannels to support high-efficient vaccine delivery and at the same time creates photothermal stress in microchannel-surrounding tissues to boost vaccination. LPD could significantly enhance pandemic influenza 2009 H1N1 vaccine immunogenicity and protective efficacy as compared with needle-based intradermal delivery in murine models. The ablative fractional laser was found to induce host DNA release, activate NLR family pyrin domain containing 3 inflammasome, and stimulate IL-1β release despite their dispensability for laser adjuvant effects. Instead, the ablative fractional laser activated MyD88 to mediate its adjuvant effects by potentiation of antigen uptake, maturation, and migration of dendritic cells. LPD also induced minimal local or systemic adverse reactions due to the microfractional and sustained vaccine delivery. Our data support the development of self-adjuvanted vaccine delivery technologies by intentional induction of well-controlled tissue stress to alert innate immune systems for more immunogenic vaccination.

## Graphical abstract

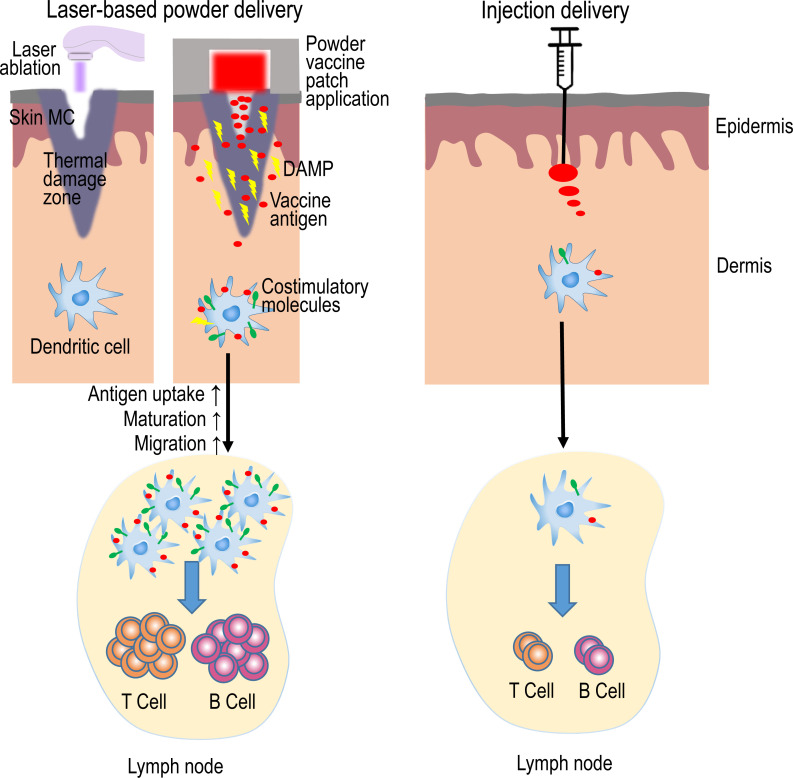

## Introduction

Vaccine delivery technologies are often designed to deliver vaccines to target tissues without causing significant tissue stress or patient discomfort. In the late 18th century, needles, lancets, and knives were used to disrupt superficial skin to deposit smallpox vaccine, the first vaccine in human history (1). Bifurcated needles were invented in 1965 to hold approximately 2.5 μL smallpox vaccine to deposit into the superficial skin by scarification (2). Bifurcated needles were the primary device used in the WHO’s smallpox eradication campaign that led to smallpox eradication in 1980 (2). Syringes and needles were first fabricated in the mid–19th century, and their manufacturing and design were gradually improved over time. Disposable syringes and needles were fabricated in the mid–20th century to reduce needle reuse–associated disease dissemination and have since been the major device used in vaccine delivery. Due to the convenience of intramuscular (IM) injection, the majority of vaccines have been injected into the muscular tissue (3). To overcome some drawbacks of needle-based injection delivery, such as needlestick injuries and sharps waste, needle-free Jet Injectors (PharmaJet, Bioject) were developed to deliver high-speed fine-stream liquid vaccines into the muscular tissue (4–7).

Skin is recognized as a highly immunogenic site for vaccine delivery (8). Intradermal (ID) delivery of rabies, hepatitis B virus surface antigen, and influenza vaccines induced more potent immune responses than IM delivery (9–11). To overcome the technical challenges of vaccine delivery to the thin dermal tissue, novel ID delivery accessories or devices were developed that included ID adapters, MicronJet600, and ID microinjection systems (7, 12–15). ID adapters and MicronJet600 are used in conjunction with traditional syringes for accurate ID vaccine delivery (13, 15). ID microinjection systems are stand-alone ID delivery devices approved for influenza vaccine delivery to save vaccine doses (12). Besides these novel ID delivery devices, dissolving microneedles are under active development for transdermal influenza vaccination (16). A recent clinical trial found dissolving microneedles applied by health care workers and patients elicited similar immune responses, supporting self-administration of dissolving microneedle-based influenza vaccines (17).

These novel delivery devices use ultrafine and short needles or completely eliminate needle use for more patient-compliant vaccine delivery (4–7, 12–16). Yet, the novel ID delivery devices induce comparable immune responses to needle-based ID delivery, and the novel transdermal and IM delivery devices induce comparable immune responses to needle-based IM delivery (4, 12, 13, 16). Vaccine adjuvants can be used in conjunction with these devices to boost vaccination. Yet, only a few adjuvants (e.g., Alum, MF59, AS04) are approved for human use due to the safety concern (18, 19). Almost all adjuvants are approved to boost IM vaccination and have a high risk to induce significant local reactions following ID or transdermal delivery due to their high reactogenicity (13, 14, 20). Besides local safety concern, ID delivery of vaccine/adjuvant mixtures by hollow microneedles or ID microinjection systems may experience increased hydraulic resistance due to the reduced needle size (21). We are also facing formulation challenges to incorporate adjuvants into transdermal delivery technologies, such as dissolving microneedles, considering the drying process will cause loss of Alum and AS04 adjuvant potency and is not compatible with oil-in-water emulsion adjuvants (MF59 and AS03). Liposome-based AS01 adjuvant may be lyophilized but faces challenges on how to prevent liposome fusion and adjuvant leakage during drying. IM delivery of Alum and emulsion adjuvant-admixed vaccines via needle-free Jet Injectors may experience increased viscosity and injection resistance. In addition, generation of high-speed liquid vaccine streams through fine nozzles within needle-free Jet Injectors may disrupt the microstructures of emulsion and liposome-based adjuvants (e.g., MF59, AS03, and AS01) and cause loss of adjuvanticity.

Pathogen-associated molecular pattern–based (PAMP-based) adjuvants, such as monophosphoryl lipid A (MPL), directly activate innate immune systems by binding to pattern recognition receptors (PRRs), while non–PAMP-based adjuvants, such as Alum and MF59, induce release of damage-associated molecular patterns (DAMPs) to activate innate immune systems. DAMPs are a group of endogenous molecules sequestered from immune system recognition under physiological conditions and can release under tissue stress to alert innate immune systems. Common types of DAMPs include host DNA, ATP, uric acid, and high mobility group box 1 (22). Alum and MF59 adjuvants were found to stimulate tissue stress and release of uric acid and ATP to enhance vaccine-induced immune responses (23, 24). Vaccine delivery technologies, if designed to stimulate DAMP release, may augment vaccine-induced immune responses without incorporation of external adjuvants.

This study introduces a prototype vaccine delivery platform, called laser-based powder delivery (LPD), which takes advantage of an aesthetic laser to generate skin microchannels (MCs) to support high-efficient vaccine delivery and at the same time create photothermal stress in MC-surrounding tissues to boost vaccination. Using ovalbumin (OVA) and influenza pandemic 2009 H1N1 (pdm09) vaccine as models, we found LPD could induce more potent immune responses than needle-based ID delivery in murine models. Further studies found LPD could significantly increase antigen uptake, maturation, and migration of dendritic cells (DCs) in skin and draining lymph nodes (LNs). Molecular adjuvantation mechanisms were also explored, and we found MyD88 played a crucial role in laser adjuvant effects, while host DNA, NLR family pyrin domain containing 3 (NLRP3) inflammasome, and IL-1β mainly mediated laser-induced local inflammation.

## Results

### Ablative fractional laser potentiates ID OVA immunization with Th2 differentiation.

**Figure 1:**
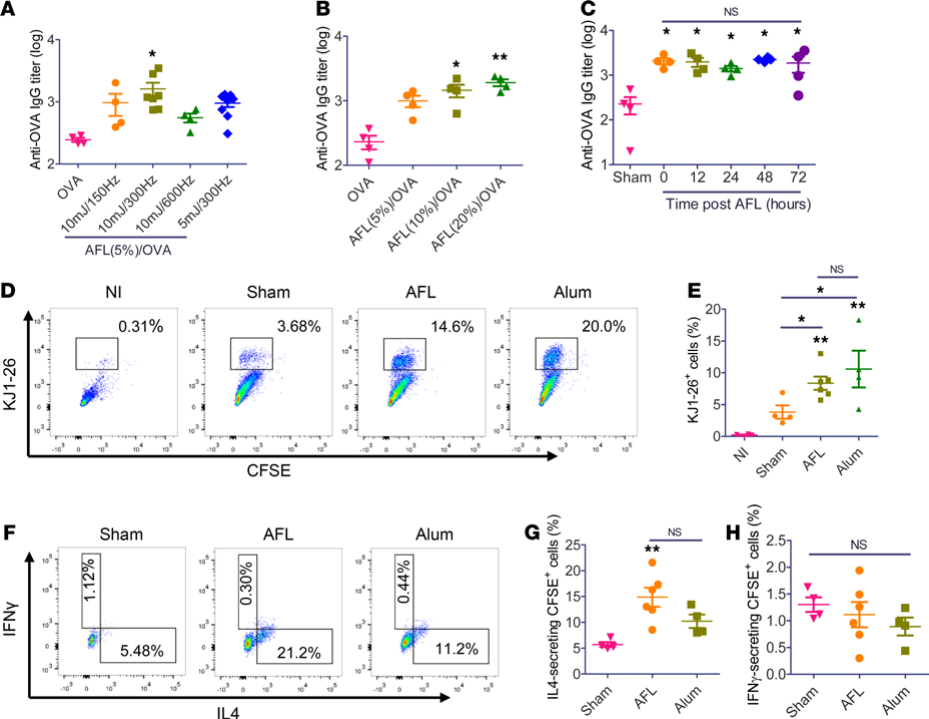
AFL boosts OVA immunization. (**A**) BALB/c mice were exposed to AFL at different laser settings followed by ID injection of 10 µg OVA right after treatment in **A** and **B** or at different time points in **C**. OVA injection into sham-treated skin served as control. Laser parameter(s) were fixed at 5% in **A**, 10 mJ/300 Hz in **B**, and 10 mJ/10%/300 Hz in **C**. Serum anti-OVA antibody titer was measured 3 weeks after immunization. (**D** and **E**) CD4^+^ T cells were purified from DO11.10 mice, stained with CFSE, and adoptively transferred to BALB/c mice followed by ID delivery of 10 μg OVA into AFL- or sham-treated skin or ID delivery of 10 μg OVA in the presence of Alum adjuvant, or left nonimmunized (NI). Draining LNs were harvested 4 days later followed by single-cell suspension preparation, immunostaining, and flow cytometry analysis. Representative dot plots about percentage of KJ1-26^+^ cells in CD4^+^ T cells (**D**). Percentage of KJ1-26^+^ cells in CD4^+^ T cells of different groups (**E**). Gating strategies are shown in Supplemental Figure 2A. (**F**–**H**) LN cells prepared in **D** and **E** were stimulated with OVA_323–339_ peptide overnight followed by immunostaining and flow cytometry analysis. Representative dot plots showing percentage of IL-4– and IFN-γ–secreting cells in KJ1-26^+^ CD4^+^ T cells (**F**). Percentage of IL-4– and IFN-γ–secreting cells in KJ1-26^+^ CD4^+^ T cells are shown in **G** and **H**, respectively. Gating strategies are shown in Supplemental Figure 2B. *n* = 4–8 (**A**–**C**), *n* = 4–6 (**D**–**H**). One-way ANOVA with Tukey’s multiple-comparison test was used to compare differences between groups (**A** and **B**). One-way ANOVA with Newman Keuls multiple-comparison test was used to compare differences (**C**, **E**, **G**, and **H**). *, *P* < 0.05; **, *P* < 0.01; NS, not significant.

Aesthetic ablative fractional laser (AFL, Lumenis UltraPulse) was used to generate skin MCs with substantial photothermal stress in surrounding tissues. Potential adjuvant effects of AFL to boost ID OVA immunization were explored first. Considering laser adjuvant effects might be affected by laser parameters, we first fixed laser percentage coverage at 5% and varied laser energy and repetition rate. As compared with OVA immunization alone, prior AFL treatment at 10 mJ energy and 300 Hz repetition rate but not in other laser conditions significantly increased anti-OVA antibody titer (Figure 1A). Impact of laser percentage coverage, an indicator of percentage skin surface exposed to fractional laser treatment, on laser adjuvant effects was then explored. Increase of laser percentage coverage from 5% to 10% and 20% significantly increased anti-OVA antibody titer (Figure 1B). Considering AFL at 20% but not 10% coverage induced skin shrinkage (Supplemental Figure 1; supplemental material available online with this article; https://doi.org/10.1172/jci.insight.144627DS1), AFL at 10 mJ energy, 10% coverage, and 300 Hz repetition rate was used in following studies. We found laser adjuvant effects could persist at local treatment sites for at least 3 days. As shown in Figure 1C, delayed OVA delivery into AFL-treated skin as long as 3 days after AFL treatment induced similar anti-OVA antibody titer to instant OVA delivery and significantly higher anti-OVA titer as compared with OVA delivery into sham-treated skin. This study supported instant vaccine delivery after AFL treatment for the convenience of vaccination.

Th cells provide crucial help for antigen-specific B cell differentiation and isotype switching to secrete specific antibodies. Next, we evaluated whether delivery of OVA into AFL-treated skin could enhance OVA-specific Th cell differentiation. To this end, OVA-specific CD4^+^ T cells were purified from DO11.10 mice, labeled with carboxyfluorescein succinimidyl ester (CFSE), and adoptively transferred to BALB/c mice followed by ID OVA delivery into AFL- or sham-treated skin or ID OVA delivery in the presence of Alum adjuvant 24 hours later. Draining LNs were collected 4 days later, and expansion of adoptively transferred CD4^+^ T cells (i.e., KJ1-26^+^ cells) was analyzed. As shown in Figure 1, D and E, a significantly higher percentage of KJ1-26^+^ cells was found in AFL and Alum groups as compared with sham group. No significant difference in percentage of KJ1-26^+^ cells was found between AFL and Alum groups (Figure 1E). Th cells can be differentiated into Th1 and Th2 cells, which secrete typical IFN-γ and IL-4 cytokines, respectively, and promote B cell isotype switching to IgG2 and IgG1, respectively (25). To explore which type of Th cells AFL stimulated, LN cells were stimulated with OVA_323–339_ followed by intracellular cytokine staining and flow cytometry analysis. As shown in Figure 1, F–H, AFL significantly increased percentage of IL-4– but not IFN-γ–secreting KJ1-26^+^ cells, while Alum failed to significantly increase percentage of IL-4– or IFN-γ–secreting KJ1-26^+^ cells. Division of adoptively transferred CFSE-labeled KJ1-26^+^ cells was also evaluated following in vitro stimulation. As shown in Supplemental Figure 3, A and B, significant division of CFSE-labeled KJ1-26^+^ cells was observed in AFL and Alum groups with barely visible peak of nondivided cells. In contrast, a mild division of CFSE-labeled KJ1-26^+^ cells was observed in the sham group with clearly visible peak of nondivided cells (Supplemental Figure 3, A and B). Frequency of KJ1-26^+^ cells after in vitro stimulation showed a similar trend to that before stimulation (Supplemental Figure 3C and Figure 1E). Similar results were also observed after adoptive transfer of OT-II T cells in C57BL/6 mice followed by the different OVA immunizations. As shown in Supplemental Figure 4, A and B, a significantly higher percentage of CFSE^+^ cells were observed in AFL and Alum groups as compared with the sham group. No significant difference in percentage of CFSE^+^ cells was found between AFL and Alum groups (Supplemental Figure 4B). Percentage of IL-4–secreting but not IFN-γ–secreting CFSE^+^ cells was significantly increased in AFL and Alum groups as compared with that in the sham group (Supplemental Figure 4, C and D). These above results indicated AFL potentiated ID OVA immunization with Th2 differentiation.

### LPD enhances OVA and pdm09 vaccination.

**Figure 2:**
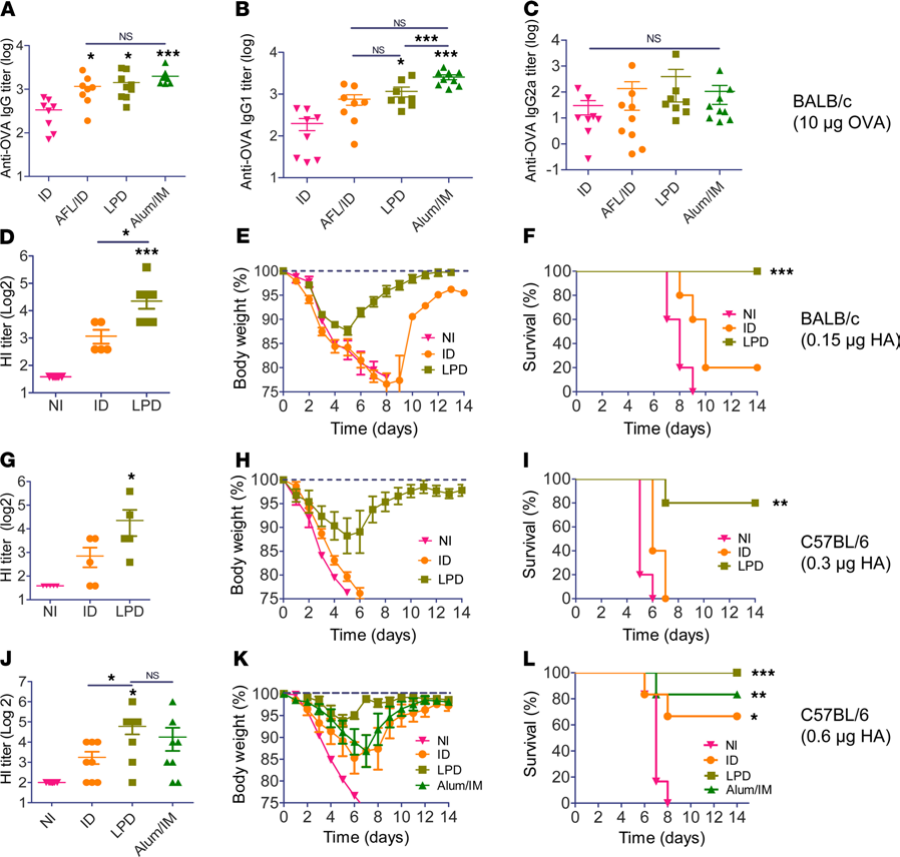
LPD boosts OVA and pdm09 vaccination. (**A**–**C**) BALB/c mice were subjected to LPD or ID delivery of OVA or ID delivery of OVA into AFL-treated skin, or IM delivery of OVA in the presence of Alum adjuvant (Alum/IM). Serum anti-OVA IgG (**A**) and subtype IgG1 (**B**) and IgG2a antibody titers (**C**) were measured 3 weeks later. (**D**–**F**) BALB/c mice were subjected to LPD or ID delivery of 0.15 μg pdm09 vaccine or left nonimmunized (NI). Serum hemagglutination inhibition (HI) titer was measured 3 weeks later (**D**). Mice were challenged with 10 × LD_50_ of mouse-adapted pdm09 viruses. Percentage of body weight loss (**E**) and survival (**F**) were monitored daily for 14 days. (**G**–**I**) C57BL/6 mice were subjected to LPD or ID delivery of pdm09 vaccine or left NI. Serum HI titer was measured 3 weeks later (**G**). Mice were then challenged with 10× LD_50_ of mouse-adapted pdm09 viruses. Percentage of body weight loss (**H**) and survival (**I**) were monitored daily for 14 days. (**J**–**L**) C57BL/6 mice were subjected to LPD or ID delivery of pdm09 vaccine, or IM delivery of pdm09 vaccine in the presence of Alum adjuvant (Alum/IM), or left nonimmunized (NI). Serum HI titer was measured 3 weeks later (**J**). Mice were challenged with 10 × LD_50_ of mouse-adapted pdm09 viruses, and percentage of body weight loss (**K**) and survival (**L**) were similarly monitored daily for 14 days. *n* = 8–9 in **A**–**C**, *n* = 5–10 in **D**–**F**, *n* = 5 in **G**–**I**, and *n* = 8 in **J**–**L**. One-way ANOVA with Newman-Keuls multiple-comparison test was used to compare differences between groups (**A**–**D**, **G**, and **J**). Log-rank test with Bonferroni’s correction was used to compare differences of survival between LPD and ID groups (**F**, **I**, and **L**). *, *P* < 0.05; **, *P* < 0.01; ***, *P* < 0.001.

We recently found AFL-generated skin MCs enabled high-efficient and sustained delivery of biologics drugs in a powder form (26). Potent adjuvant effects of AFL identified in above studies hinted LPD might possess potent adjuvant effects to boost vaccination. We first compared LPD with needle-based ID delivery and Alum-adjuvanted IM delivery using OVA as a model antigen. As shown in Figure 2A, LPD induced significantly higher anti-OVA antibody titer as compared with ID delivery and comparable anti-OVA antibody titer to Alum-adjuvanted IM delivery. LPD also induced similar antibody titer to AFL-adjuvanted ID delivery (Figure 2A). This study indicated LPD possessed potent adjuvant effects to boost OVA immunization. Furthermore, LPD mainly enhanced anti-OVA IgG1 but not IgG2a antibody titer, in line with the induction of Th2 differentiation by AFL (Figure 2, B and C).

Next, pdm09 vaccine was used to compare the relative immunogenicity of LPD with needle-based ID delivery. As shown in Figure 2D, LPD but not ID delivery significantly increased serum HI titer. Following lethal viral challenges, mice in the LPD group lost a maximum of 12% body weight on day 5, while the majority of mice in ID and NI groups either died or lost at least 25% body weight (humane endpoint) in 8 days (Figure 2E). Mice in the LPD group started to recover on day 6 and completely recovered to their original body weight on day 14 (Figure 2E). LPD conferred 100% protection, while ID delivery only conferred 20% protection (Figure 2F). This study indicated LPD was more immunogenic than needle-based ID delivery for pdm09 vaccine delivery. More immunogenic LPD than ID delivery was also observed in C57BL/6 mice. As shown in Figure 2G, LPD but not ID delivery significantly increased serum HI titer. Mice in NI and ID groups either died or lost at least 25% body weight in 5–6 days, while mice in the LPD group lost a maximum of 12% body weight on day 5 (Figure 2H). Mice in the LPD group started to recover on day 6 and recovered to 98% of their original body weight on day 14 (Figure 2H). LPD conferred 80% protection, while ID delivery failed to confer protection (Figure 2I).

ID influenza vaccine induces significant HI titer and protection in clinics (12, 14). In the above studies, ID pdm09 vaccine induced inferior protection. To more closely simulate clinical conditions, we increased pdm09 vaccine dose to allow ID delivery to induce marked protection and then compared relative immunogenicity of LPD with ID delivery. Alum-adjuvanted IM delivery was also included for comparison, although Alum adjuvant was found to be ineffective to boost influenza vaccination (27–30). As shown in Figure 2J, LPD but not ID delivery or Alum-adjuvanted IM delivery significantly increased serum HI titer. Following lethal viral challenges, mice in the LPD group lost a maximum of 6% body weight on day 5, while mice in ID delivery and Alum-adjuvanted IM delivery groups lost a maximum of 15% and 14% body weight, respectively, on day 7 (Figure 2K). Similar body weight loss was observed on each day between ID delivery and Alum-adjuvanted IM delivery, while significantly less body weight loss was observed on days 6–8 in LPD as compared with ID delivery and on day 7 in LPD as compared with Alum-adjuvanted IM delivery (Supplemental Table 2). Mice in the NI group either died or lost at least 25% body weight in 8 days (Figure 2K). Mice with LPD recovered to 98% of their original body weight on day 7, while mice with ID delivery and Alum-adjuvanted IM delivery recovered to the same percentage of the original body weight on days 13 and 11, respectively (Figure 2K). LPD conferred 100% protection, while ID delivery and Alum-adjuvanted IM delivery conferred 67% and 83% protection, respectively (Figure 2L). The above body weight change indicated superiority of LPD to ID delivery and Alum-adjuvanted IM delivery for pdm09 vaccine delivery. Our data also indicated Alum adjuvant was not very effective in boosting pdm09 vaccination.

Local safety of LPD was also explored in the above studies. Transepidermal water loss (TEWL) was used to monitor skin integrity. AFL generated visible skin MCs with significantly increased TEWL (Supplemental Figure 5), hinting loss of skin integrity. TEWL of AFL-treated skin significantly decreased on day 2 after patch removal but was still higher than the baseline level, hinting partial recovery of skin integrity (Supplemental Figure 5B). TEWL returned to the baseline level 2 days after patch removal (Supplemental Figure 5B), hinting complete recovery of skin integrity. An array of skin dents, reminiscent of skin MCs, were visible on day 4 but disappeared on day 10 (Supplemental Figure 5A), indicating complete recovery of skin morphology. Systemic safety of LPD was also evaluated by measurement of rectal temperature. ID delivery but not LPD or Alum-adjuvanted IM delivery significantly increased rectal temperature at 24 hours (Supplemental Figure 6A). Rectal temperature returned to the baseline level at 48 hours in all groups (Supplemental Figure 6B). These results indicated good local and systemic safety of LPD for pdm09 vaccine delivery.

### LPD enhances DC function.

**Figure 3:**
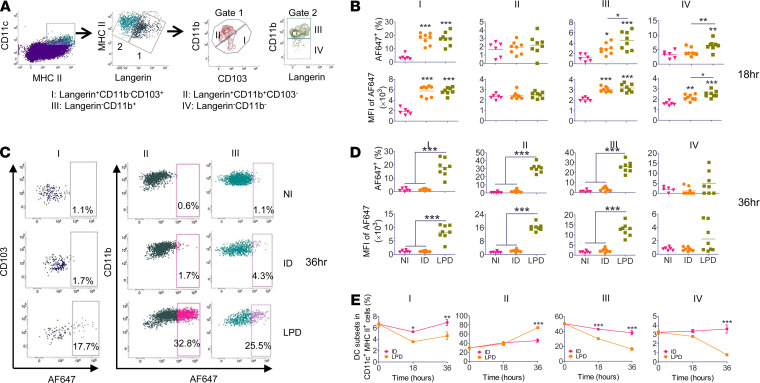
LPD enhances local antigen uptake. C57BL/6 mice were subjected to LPD or ID delivery of AF647-OVA or left NI. Skin was collected 18 or 36 hours later. Single-cell suspensions were prepared followed by immunostaining and flow cytometry analysis. (**A**) Representative dot plots showing gating strategies of different DC subsets in the skin. More gating strategies are shown in Supplemental Figure 2C. (**B**) Percentage of AF647^+^ cells and MFI of AF647 in different DC subsets in the skin at 18 hours. (**C**) Representative dot plots showing percentage of AF647^+^ cells in different DC subsets in the skin at 36 hours. (**D**) Percentage of AF647^+^ cells and MFI of AF647 in different DC subsets in the skin at 36 hours. (**E**) Changes of individual DC subset levels in the skin following LPD and ID delivery of AF647-OVA. *n* = 6–8. One-way ANOVA with Tukey’s multiple-comparison test was used to compare differences between groups (**B** and **D**). Two-way ANOVA with Bonferroni’s posttest was used to compare differences between groups at different time points (**E**). *, *P* < 0.05; **, *P* < 0.01; ***, *P* < 0.001.

DCs play a pivotal role in bridging innate and adaptive immunity (31). Vaccine adjuvants often act on DCs to enhance vaccine-induced immune responses (32, 33). Next, antigen uptake and maturation of DCs were compared following LPD and ID delivery of Alexa Fluor 647–conjugated OVA (AF647-OVA) at 18 and 36 hours. According to reports (34, 35), skin DCs were divided into 4 subsets: Langerin^+^CD11b^–^CD103^+^ (I), Langerin^+^CD11b^+^CD103^–^ (II) or Langerhans cells (LCs), Langerin^–^CD11b^+^ (III), and Langerin^–^CD11b^–^ DCs (IV) (Figure 3A). LPD and ID delivery similarly increased percentage of AF647-OVA^+^ cells in DC subset I and MFI of AF647 in DC subsets I and III at 18 hours (Figure 3B). LPD more significantly increased percentage of AF647-OVA^+^ cells in DC subsets III and IV and MFI of AF647 in DC subset IV as compared with ID delivery at 18 hours (Figure 3B). Significantly higher percentage of AF647-OVA^+^ cells and MFI of AF647 were found in DC subsets I–III in LPD as compared with ID delivery at 36 hours, while similar levels of AF647-OVA^+^ cells and MFI of AF647 were found in all DC subsets between ID and NI groups at this time point (Figure 3, C and D).

Skin DC subset levels were also monitored. Skin DC subset I levels were more significantly reduced in LPD as compared with ID delivery at 18 hours (Figure 3E). Skin DC subset I levels completely recovered at 36 hours in ID delivery and partially recovered in LPD at this time point (Figure 3E). Skin DC subset III levels gradually declined in both deliveries and more significantly in LPD as compared with ID delivery (Figure 3E). Skin DC subset IV levels were slightly reduced at 18 hours and drastically reduced at 36 hours in LPD, while skin DC subset IV levels showed no significant change in ID delivery at both time points (Figure 3E). Interestingly, skin DC subset II levels similarly increased at 18 hours in both deliveries and more significantly increased in LPD as compared with ID delivery at 36 hours (Figure 3E). Considering skin DC subsets (I, III, and IV) belong to dermal DCs, the above data indicated more significant reduction of dermal DCs in LPD as compared with ID delivery.

**Figure 4:**
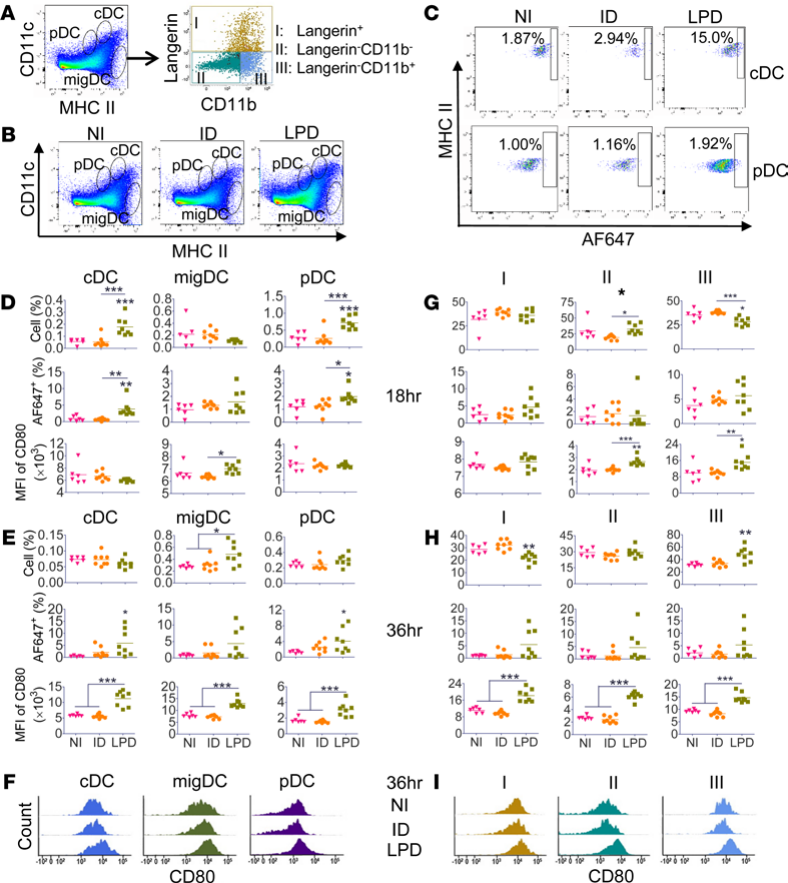
LPD enhances antigen uptake and maturation of DCs in draining LNs. C57BL/6 mice were subjected to LPD or ID delivery of AF647-OVA or left NI. Draining LNs were collected 18 and 36 hours later. Single-cell suspensions were prepared followed by immunostaining and flow cytometry analysis. (**A**) Representative dot plot showing gating strategies of different DC and migDC subsets. More gating strategies are shown in Supplemental Figure 2D. (**B**) Representative dot plots showing percentage of different DC subsets at 18 hours. (**C**) Representative dot plots showing percentage of AF647^+^ cells in cDC and pDC subsets at 18 hours. (**D** and **E**) Percentage of different DC subsets, percentage of AF647^+^ cells, and MFI of CD80 in different DC subsets at 18 (**D**) and 36 hours (**E**), respectively. (**F**) Representative histogram of CD80 levels in different DC subsets at 36 hours. (**G** and **H**) Percentage of different migDC subsets, percentage of AF647^+^ cells, and MFI of CD80 in different migDC subsets at 18 (**G**) and 36 hours (**H**), respectively. (**I**) Representative histogram of CD80 levels in different migDC subsets at 36 hours. *n* = 6–8. One-way ANOVA with Newman-Keuls multiple-comparison test was used to compare differences between groups. *, *P* < 0.05; **, *P* < 0.01; ***, *P* < 0.001.

Antigen uptake and maturation of DCs in draining LNs were then explored. DCs in draining LNs were divided into conventional DCs (cDCs), migratory DCs (migDCs), and plasmacytoid DCs (pDCs) based on the relative expression of MHC II and CD11c, and migDCs were further divided into 3 subsets based on the relative expression of Langerin and CD11b (Figure 4A). Increased cDC and pDC levels were observed in LPD as compared with ID delivery at 18 hours (Figure 4B). Higher percentage of AF647^+^ cDCs and pDCs were found in LPD as compared with ID delivery at 18 hours (Figure 4C). LPD significantly increased cDC and pDC levels as well as AF647^+^ cDC and pDC levels at 18 hours as compared with ID delivery (Figure 4D). LPD also significantly increased MFI of CD80 as compared with ID delivery at 18 hours (Figure 4D). At 36 hours, LPD but not ID delivery significantly increased migDC levels as well as AF647^+^ cDC and pDC levels (vs. NI group). Interestingly, LPD but not ID delivery significantly increased MFI of CD80 in all DC subsets at 36 hours (Figure 4, E and F). These results indicated LPD could significantly increase antigen^+^ cDC and pDC levels at both time points and antigen^+^ migDC levels and maturation of all DC subsets at the later time point. Next, we tried to identify specific migDC subset(s) responsible for the enhanced migDC function in LPD (Figure 4, D and E). At 18 hours, increased MFI of CD80 in migDCs in LPD (Figure 4D) was mainly contributed by migDC subsets II and III (Figure 4G). At 36 hours, increased migDC levels in LPD (Figure 4E) were mainly contributed by migDC subset III (Figure 4H), which had the same phenotype as skin DC subset III (Figure 3A). This result was in line with the more significant reduction of the most abundant skin DC subset III in LPD as compared with ID delivery at this time point (Figure 3E). We further found increased MFI of CD80 in migDCs in LPD (Figure 4E) was contributed by all migDC subsets (Figure 4, H and I).

### AFL induces strong local inflammation.

**Figure 5:**
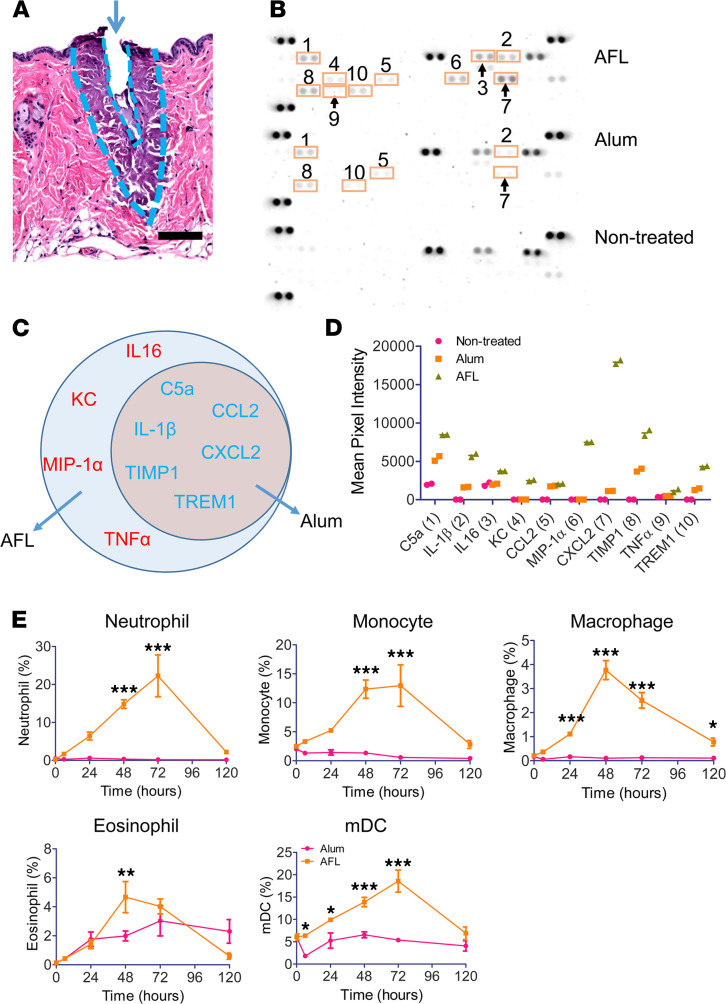
AFL induces strong local inflammation. (**A**) Lateral back skin of BALB/c mice was exposed to AFL, dissected right after treatment, and subjected to histological analysis. Representative skin MC (arrow) and thermal damage zone are shown. Edges of skin MC and thermal damage zone were marked with dashed blue lines. Scale bar: 100 μm. (**B**–**D**) Lateral back skin of BALB/c mice was exposed to AFL, intradermally injected with Alum adjuvant, or left nontreated. Skin was dissected 24 hours later. Total proteins were extracted, pooled (*n* = 3), and subjected to membrane immunoblotting analysis of cytokine levels. Representative immunoblotting images (**B**). The commonly and differentially stimulated cytokines by AFL and ID Alum adjuvant (**C**). Mean pixel intensities of different cytokines in **B** were measured by ImageJ (NIH) and compared (**D**). The duplicate cytokine spots within the rectangles in **B** are labeled with numbers to indicate increased cytokine levels after treatment. The corresponding cytokine name can be found in **D** (*x* axis). (**E**) Innate immune cell recruitment after AFL treatment and ID Alum adjuvant injection. Lateral back skin of mice was exposed to AFL or intradermally injected with Alum adjuvant. Skin was excised at different time points. Single-cell suspensions were prepared followed by immunostaining and flow cytometry analysis. Percentage of neutrophils, monocytes, macrophages, eosinophils, and mDCs in total skin cells is shown. Gating strategies are shown in Supplemental Figure 2E. *n* = 4. Two-way ANOVA with Bonferroni’s posttest was used to compare differences between groups at different time points (**E**). *, *P* < 0.05; **, *P* < 0.01; ***, *P* < 0.001.

We believe enhanced DC function in LPD was mainly due to the activated innate immunity rather than the 12-hour sustained vaccine delivery (26). In support, LPD of OVA induced comparable anti-OVA antibody titer to ID OVA delivery into AFL-treated skin (Figure 2A). A recent study found sustained vaccine delivery without adjuvants failed to induce more potent immune responses as compared with instant vaccine delivery (36). Thus, we mainly focused on exploiting thermal tissue stress and the activation of innate immune systems to delineate the observed laser adjuvant effects. As shown in Figure 5A, AFL induced substantial photothermal stress in MC-surrounding tissues. Thermal damage zones were estimated to be 13.5-fold larger in volume than skin MCs because of the 3-fold difference in diameter and 1.5-fold difference in depth between the 2 cone-shaped structures (Figure 5A). Thermal damage was expected to elicit acute immune responses to clear damaged tissues and restore tissue homeostasis. Alum adjuvant was reported to also induce tissue damage and activate acute immune responses (37). Here, we compared local innate immune responses induced by AFL and ID Alum adjuvant. Skin cytokine levels were first compared 24 hours after treatment. As shown in Figure 5B, AFL induced marked expression of 10 out of 40 cytokines, including complement component 5a (C5a), IL-1β, IL-16, keratinocyte chemoattractant (KC), CC motif chemokine ligand 2 (CCL2), macrophage inflammatory protein-1α (MIP-1α), CXC motif chemokine ligand 2 (CXCL2), tissue inhibitors of metalloproteinase 1 (TIMP1), tumor necrosis factor–α (TNF-α), and triggering receptor expressed on myeloid cells 1 (TREM1). C5a plays a key role in neutrophil and monocyte extravasation. KC and CXCL2 are neutrophil chemoattractants and MIP-1α recruits polymorphonuclear leukocytes. CCL2 is a monocyte chemoattractant and IL-16 recruits CD4-expressing T cells, monocytes, eosinophils, and DCs. TIMP1 plays a role in extracellular matrix remodeling. TNF-α and IL-1β are potent inflammatory cytokines. TREM1 plays an important role in amplification of inflammation. Interestingly, 6 of the 10 cytokines (C5a, IL-1β, CCL2, CXCL2, TIMP1, and TREM1) stimulated by AFL were also induced by Alum adjuvant (Figure 5B). Six cytokines stimulated by Alum adjuvant were all induced by AFL (Figure 5C). AFL also induced higher levels of cytokine expression than ID Alum adjuvant (Figure 5D).

Innate immune cell recruitment was then explored. AFL vigorously recruited neutrophils, monocytes, macrophages, eosinophils, and myeloid DCs (mDCs), while ID Alum adjuvant only significantly recruited eosinophils (Figure 5E). Skin neutrophil levels in LPD steadily increased to peak levels at 22%, while skin neutrophil levels in the Alum group remained below 1% at all time points (Figure 5E). AFL significantly increased skin monocyte and macrophage levels to peak at 13% and 3.8%, respectively (Figure 5E). No significant increase of skin monocyte and macrophage levels was observed in the Alum group (Figure 5E). Both AFL and Alum increased skin eosinophil levels (Figure 5E). AFL induced transient recruitment of eosinophils with skin eosinophil levels approaching the baseline level at 120 hours (Figure 5E). ID Alum adjuvant increased and maintained skin eosinophil levels above 2% after 48 hours (Figure 5E). AFL steadily increased skin mDC levels to peak at 19%, while ID Alum adjuvant failed to significantly increase skin mDC levels (Figure 5E). Our data support more potent recruitment of innate immune cells by AFL as compared with ID Alum adjuvant.

### AFL stimulates DNA and IL-1β release and activates NLRP3 inflammasome despite their dispensability for laser adjuvant effects.

**Figure 6:**
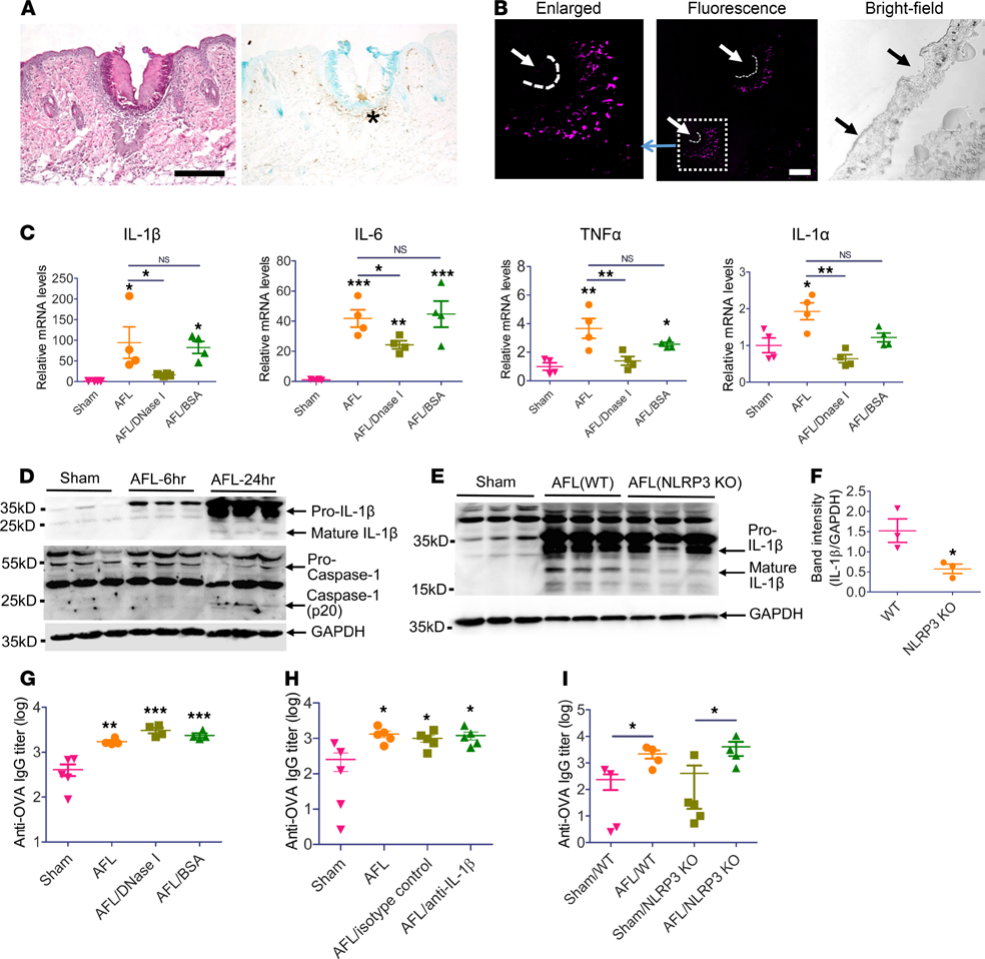
Laser stimulates DNA and IL-1β release and activates NLRP3 inflammasome despite their dispensability for laser adjuvant effects. (**A**) Skin of C57BL/6 mice was exposed to AFL and excised 24 hours later for side-by-side histological (left) and apoptosis analyses (right). Brown indicates apoptosis signal (*). Scale bar: 100 μm. (**B**) Skin of C57BL/6 mice was exposed to AFL followed by subcutaneous injection of DRAQ7. Skin was excised 30 minutes later, cryosectioned, and imaged. Dashed curved line: edges of skin MCs. Arrows: skin MCs. Scale bar: 100 μm. (**C**) C57BL/6 mice were exposed to AFL followed by ID injection of DNase I (2500 units) or BSA (280 μg) or exposed to AFL or sham treatment. Skin was dissected 6 hours later for real-time PCR analysis. (**D**) Skin of BALB/c mice was exposed to AFL or sham treatment. Skin was dissected 6 and 24 hours later for Western blotting. (**E**) WT and NLRP3-KO mice were subjected to AFL or sham treatment. Skin was dissected 24 hours later for Western blotting. (**F**) Relative band intensities of IL-1β to GAPDH were compared. (**G**) BALB/c mice were subjected to AFL treatment followed by ID injection of DNase I (2500 units) or BSA (280 μg) or subjected to AFL or sham treatment. Thirty minutes later, 10 μg OVA was intradermally injected in all groups. (**H**) C57BL/6 mice were subjected to AFL or sham treatment followed by ID injection of 10 μg OVA or subjected to AFL treatment followed by ID injection of 10 μg OVA mixed with 10 μg anti–IL-1β antibodies or isotype control. The latter 2 groups were also intraperitoneally injected with 200 μg anti–IL-1β antibodies or isotype control 1 hour before and 18 hours after immunization. (**I**) WT and NLRP3-KO mice were subjected to AFL or sham treatment followed by ID injection of 10 μg OVA. Serum anti-OVA antibody titer in **G**–**I** was measured 3 weeks later. *n* = 4 (**C**), *n* = 4–5 (**G**–**I**). One-way ANOVA with Newman-Keuls multiple-comparison test was used to compare differences between groups (**C**, **G**, and **H**). One-tailed Mann-Whitney *U* test was used to compare differences between groups (**F** and **I**). *, *P* < 0.05; **, *P* < 0.01; ***, *P* < 0.001.

AFL most likely stimulates DAMP release to potentiate vaccine-induced immune responses. Due to heat dissipation, a temperature gradient was likely to form in MC-surrounding tissues. High temperature near the center might cause instant cell deaths, while not so high temperature in the periphery might cause cell apoptosis. To explore this, an in situ apoptosis kit was used to detect fragmented DNA, a hallmark of cell apoptosis, in AFL-treated skin. As shown in Figure 6A, tissue damage was clearly visible in MC-surrounding tissues with large amounts of immune cell infiltration into the periphery but not the center of thermal damage zones at 24 hours (left). Apoptotic signals were found only in the periphery but not the center of thermal damage zones (right, Figure 6A). These results hinted AFL induced tissue necrosis in most areas of thermal damage zones. Tissue necrosis may release DAMPs to alert innate immune systems. Host DNA was among the most explored DAMPs and was found to be released after Alum adjuvant or non-AFL treatment (38, 39). To explore potential host DNA release, cell-impermeable DNA dye DRAQ7 was subcutaneously injected according to a report (39). Skin was collected 30 minutes later and subjected to cryosectioning and fluorescence imaging. As shown in Figure 6B, intense DNA staining was observed in MC-surrounding tissues but not healthy tissues farther away. Interestingly, we failed to detect DNA signals 6 or 24 hours after AFL treatment (data not shown). These results indicated AFL induced instant DNA release.

Next, we explored whether AFL-induced cytokine release was mediated by host DNA. To this end, recombinant DNase I or BSA was intradermally injected into AFL-treated skin right after AFL treatment, and cytokine gene expression was evaluated by real-time PCR. As shown in Figure 6C, AFL treatment significantly increased mRNA levels of IL-1β, IL-6, TNF-α, and IL-1α by ~94-, 42-, 4-, and 2-fold, respectively. DNase I treatment significantly reduced mRNA levels, while BSA treatment had no significant effect (Figure 6C). This result indicated DNA played a crucial role in AFL-induced IL-1β, IL-6, TNF-α, and IL-1α gene expression. AFL also increased mRNA levels of IL-10 but not IFN-α, IFN-β, or IFN-γ (Supplemental Figure 7A). AFL also significantly increased mRNA levels of chemokines, such as CCL2, CCL7, and E selectin, but not CCL12, Chemerin, or CXCL9 (Supplemental Figure 7B). Interestingly, DNA was not crucial for AFL-induced IL-10, CCL2, CCL7, and E selectin gene expression (Supplemental Figure 7).

IL-1β synthesis is tightly controlled. It is first synthesized as a pro-form and then catalyzed by Caspase-1 to become an active form (40). Next, we explored whether AFL stimulated the synthesis of the active form of IL-1β. As shown in Figure 6D, a substantial amount of pro–IL-1β was synthesized 24 hours after AFL treatment, and the active form of IL-1β could also be detected at this time point. Considering caspase-1 also exists as a pro-form and requires proteolytic cleavage via inflammasome assembly to form the active heterodimers (p10/20), we further analyzed maturation status of Caspase-1. As shown in Figure 6D, Caspase-1 (p20) band could be detected 24 hours after AFL treatment, in line with the presence of the active form of IL-1β at this time point. These results indicated AFL treatment could activate Caspase-1 to process the newly synthesized pro–IL-1β into the active form of IL-1β.

Caspase-1 activation requires inflammasome assembly (40). Inflammasomes are a group of multiprotein complexes composed of an inflammasome sensor, the adapter protein apoptosis-associated speck-like protein containing a C-terminal caspase recruitment domain, and Caspase-1 (40). NLRP3 inflammasome is one of the most explored inflammasomes and can be activated by a variety of stimuli, including double-stranded DNA (40). Next, we explored whether NLRP3 inflammasome played a role in AFL-stimulated IL-1β release. To this end, wild-type (WT) and NLRP3-KO mice were subjected to AFL treatment followed by detection of the active form of IL-1β 24 hours later. As shown in Figure 6, E and F, NLRP3-KO mice showed significantly reduced levels of the active form of IL-1β as compared with WT mice, hinting NLRP3 inflammasome played an important role in AFL-stimulated synthesis of the active form of IL-1β.

Next, we explored whether host DNA, IL-1β, and NLRP3 inflammasome played a crucial role in laser adjuvant effects. In the first experiment, mice were treated with AFL followed by ID injection of DNase I to hydrolyze released DNA and explore its impact on ID OVA immunization. As shown in Figure 6G, depletion of host DNA with DNase I had no significant impact on AFL-enhanced anti-OVA antibody production, indicating host DNA was not essential to laser adjuvant effects. In the second experiment, mice were treated with AFL followed by ID injection of neutralizing antibodies against IL-1β to explore its impact on ID OVA immunization. As shown in Figure 6H, IL-1β depletion had no significant impact on AFL-enhanced anti-OVA antibody production, indicating IL-1β was not essential to laser adjuvant effects. In the third experiment, laser adjuvant effects to boost ID OVA immunization were compared between NLRP3-KO and WT mice. As shown in Figure 6I, AFL similarly enhanced OVA-induced antibody responses in NLRP3-KO mice as in WT mice, hinting NLRP3 inflammasome was not essential to laser adjuvant effects. The in vivo OVA immunization studies indicated host DNA, IL-1β, and NLRP3 inflammasome mainly contributed to AFL-induced inflammation but not antigen-specific innate or adaptive immune responses.

### AFL activates MyD88 to mediate its adjuvant effects.

**Figure 7:**
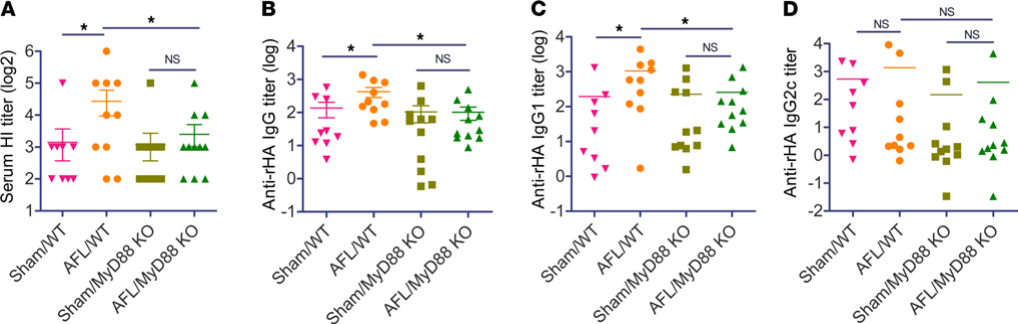
AFL activates MyD88 to mediate laser adjuvant effects. WT and MyD88-KO mice were subjected to AFL or sham treatment followed by ID injection of 0.3 μg pdm09 vaccine into AFL- or sham-treated skin. Serum HI titer (**A**), and anti-rHA IgG (**B**) and subtype IgG1 (**C**) and IgG2c antibody titers (**D**) were measured 3 weeks later. *n* = 9–11. One-tailed Student’s *t* test was used to compare differences between groups. *, *P* < 0.05.

MyD88 is a key adaptor molecule of Toll-like receptor (TLR) and IL-1 receptor (IL-1R) signaling pathways (41). MyD88 mediates adjuvant effects of the majority of PAMP-based adjuvants, such as MPL, imiquimod, and CpG (42). Interestingly, MyD88 also mediates adjuvant effects of non–PAMP-based adjuvants, such as MF59 (43) and physical radiofrequency-based adjuvant (44), and can be activated by various types of DAMPs after binding to PRRs (22). Last, we explored whether MyD88 mediated laser adjuvant effects. To this end, WT and MyD88-KO mice were subjected to AFL or sham treatment followed by ID pdm09 vaccination. As shown in Figure 7A, AFL treatment significantly increased serum HI titer in WT but not MyD88-KO mice. Similarly, AFL significantly increased anti–recombinant hemagglutinin antigen (anti-rHA) IgG titer in WT but not MyD88-KO mice (Figure 7B). These data indicated crucial roles of MyD88 in laser adjuvant effects. Furthermore, AFL significantly increased anti-rHA IgG1 but not IgG2c antibody titer in WT mice (Figure 7, C and D), in line with the induction of Th2 differentiation by AFL (Figure 1). As expected, AFL failed to significantly increase anti-rHA IgG1 or IgG2c antibody titer in MyD88-KO mice (Figure 7, C and D).

**Figure 8:**
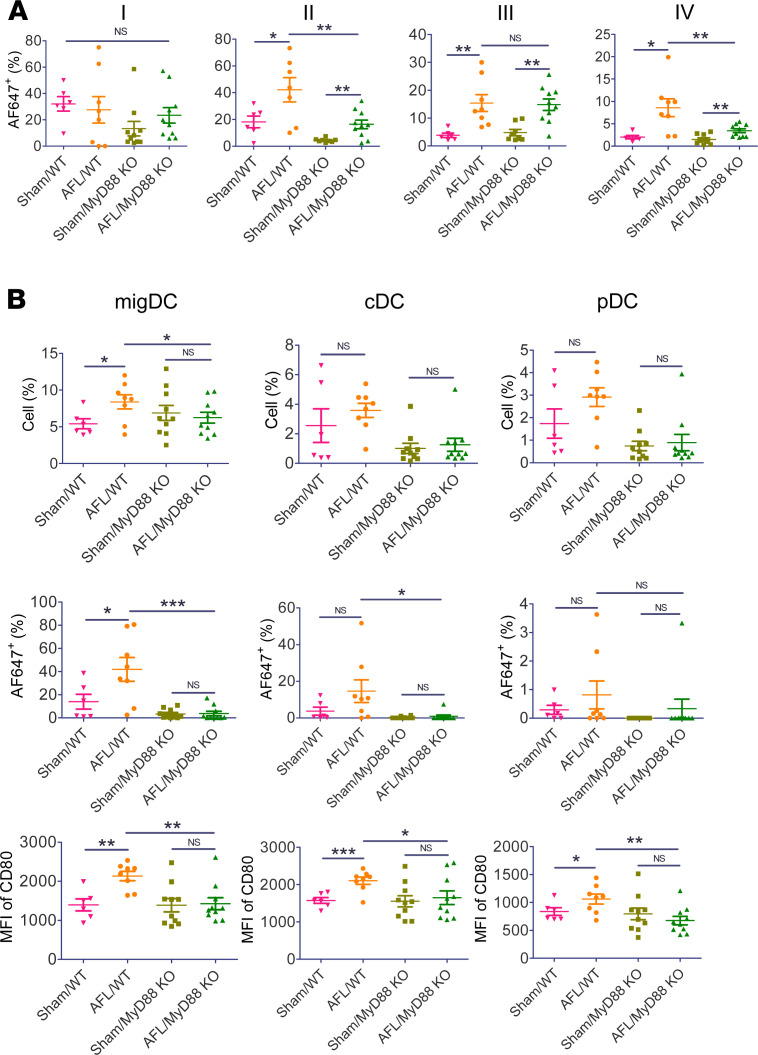
Crucial roles of MyD88 in AFL-enhanced antigen uptake and DC maturation. WT and MyD88-KO mice were subjected to AFL or sham treatment followed by ID injection of 10 μg AF647-OVA into AFL- or sham-treated skin. (**A**) Skin was harvested 24 hours later followed by single-cell suspension preparation, immunostaining, and flow cytometry analysis of percentage of AF647^+^ cells in skin DC subsets. Gating strategy is shown in Supplemental Figure 2C. (**B**) Draining LNs were harvested 24 hours later followed by single-cell suspension preparation, immunostaining, and flow cytometry analysis of percentage of DC subsets (upper), percentage of AF647^+^ cells in DC subsets (middle), and MFI of CD80 in DC subsets (lower). Gating strategies are shown in Supplemental Figure 2D. *n* = 6–10. One-tailed Student’s *t* test was used to compare differences between groups. *, *P* < 0.05; **, *P* < 0.01; ***, *P* < 0.001.

Impact of MyD88 knockout on antigen uptake and maturation of DCs was then explored. To this end, MyD88-KO and WT mice were subjected to AFL or sham treatment followed by ID injection of AF647-OVA into AFL or sham-treated skin. Antigen uptake and maturation of DC subsets in skin and draining LNs were analyzed 24 hours later. As shown in Figure 8A, AFL similarly increased percentage of AF647^+^ cells in skin DC subset III in WT and MyD88-KO mice and more significantly increased percentage of AF647^+^ cells in skin DC subsets II and IV in WT than MyD88-KO mice. Interestingly, AFL failed to significantly increase percentage of AF647^+^ cells in skin DC subset I in WT or MyD88-KO mice (Figure 8A). MFI of AF647 showed a similar pattern to percentage of AF647^+^ cells (Supplemental Figure 8A). These results indicated MyD88 contributed to more significant local antigen uptake in the AFL group.

In draining LNs, AFL significantly increased migDC levels in WT but not MyD88-KO mice (Figure 8B). AFL also significantly increased percentage of AF647^+^ cells in migDCs in WT but not MyD88-KO mice (Figure 8B). AFL had no significant impact on cDC or pDC levels or percentage of AF647^+^ cDC or pDC levels in WT or MyD88-KO mice (Figure 8B). AFL also significantly increased MFI of CD80 in all DC subsets in WT but not MyD88-KO mice (Figure 8B). MFI of CD80 in all DC subsets in the AFL/WT group was significantly higher than that in the AFL/MyD88-KO group (Figure 8B). These results indicated crucial roles of MyD88 in AFL-enhanced antigen uptake in migDCs and AFL-enhanced maturation of all DC subsets in draining LNs.

## Discussion

Vaccine delivery technologies and vaccine adjuvants are crucial considerations in vaccine development. Needle-based IM injection has been the major method of vaccine delivery in the modern era. In pursuit of more immunogenic vaccination, novel ID and transdermal delivery technologies were developed to deliver vaccines into the more immunogenic skin tissue. Yet, skin vaccination only slightly enhances vaccine-induced immune responses. Incorporation of vaccine adjuvants is promising to further enhance vaccine-induced immune responses. Yet, the majority of vaccine adjuvants are not compatible for skin delivery due to the local safety concern. We are also facing formulation challenges to incorporation of currently approved adjuvants into the novel ID or transdermal delivery technologies. This study presents LPD as a potentially novel transdermal delivery platform capable of eliciting more potent immune responses than needle-based ID delivery. LPD of OVA elicited more potent immune responses than needle-based ID delivery (Figure 2A). LPD of pdm09 vaccine elicited more potent immune responses and protection than needle-based ID delivery (Figure 2, D–L). To our knowledge, this is the first time that transdermal vaccine delivery in the absence of adjuvants induced better immune responses and protection than needle-based ID delivery.

Inherent adjuvant effects of LPD were largely due to the unique AFL. Significant adjuvant effects of AFL were elicited at relatively strong (10 mJ energy and 10% coverage) but not weak laser conditions (e.g., 2.5/5 mJ energy and 5% coverage) (26, 45, 46). Adjuvant effects of AFL were most likely due to its induction of significant photothermal stress in MC-surrounding tissues to alert innate immune systems. We found AFL could induce instant DNA release to stimulate pro–IL-1β synthesis and activate NLRP3 inflammasome to mediate mature IL-1β release, although DNA, IL-1β, and NLRP3 inflammasome were dispensable for laser adjuvant effects (Figure 6G). Instead, we found MyD88 played a crucial role in laser adjuvant effects (Figures 7 and 8). It is intriguing how MyD88 became essential to the different physical and chemical adjuvant effects. Since most DAMPs bind to TLRs (22), the association of MyD88 with most of the TLRs likely makes it an essential molecule for most DAMP-based adjuvants. Our studies found DNA mediated synthesis of some but not all cytokines (Supplemental Figure 7), indicating other DAMPs might be released under tissue necrosis. More studies would be needed to identify other DAMP(s) and downstream signaling pathways to fully understand the molecular adjuvantation mechanisms of AFL.

LPD significantly increased antigen uptake and maturation of DCs in skin and/or draining LNs (Figure 3 and 4). LPD also promoted DC migration from skin to draining LNs as evidenced by significantly increased migDC levels in LPD as compared with ID delivery at 36 hours (Figure 4E). Considering migDCs in draining LNs were originated from peripheral tissues (47), increased migDC levels in LPD were most likely due to increased egress from the skin. This was consistent with more significant reduction of dermal DC levels in LPD than ID delivery (Figure 4E). We believe increased epidermal LCs in LPD at 36 hours were not due to increased survival, considering thermal heating likely affects all skin cell types equally. The increased epidermal LCs were rather due to the significant migration of dermal DCs to draining LNs in consideration of the slow migration and turnover of LCs under inflammation (48, 49). AFL followed by ID immunization also significantly increased antigen uptake, maturation, and migration of DCs from skin to draining LNs (Figure 8). AFL was found to also significantly increase skin mDC levels (Figure 5E). Increased mDCs might be differentiated from infiltrating monocytes and contribute to increased antigen uptake and migration to draining LNs in LPD, in which vaccine antigens were slowly released over 12 hours (26). Increased DC function is expected to contribute significantly to the cellular adjuvantation mechanisms of LPD and AFL due to its crucial roles in bridging innate and adaptive immunity.

Significant similarities were found between AFL and Alum adjuvant. Both induced tissue necrosis and eosinophil infiltration (Figure 5E) (23). Both stimulated pro–IL-1β synthesis (signal 1) and activated NLRP3 inflammasome and Caspase-1 (signal 2) to mediate the active IL-1β release (Figure 6, D and E), in line with the 2-signal model of IL-1β production (40). Moreover, NLRP3 inflammasome and IL-1β were dispensable for AFL or Alum adjuvant effects (Figure 6, H and I) (50). Both induced Th2-biased immune responses and isotype switching to IgG1 (Figures 1 and 2). Despite these similarities, AFL showed disparities from Alum adjuvant in that AFL vigorously recruited neutrophils, monocytes, macrophages, and mDCs, while ID Alum adjuvant failed to significantly recruit these innate immune cells (Figure 5E). Furthermore, AFL stimulated MyD88 to mediate its adjuvant effects (Figure 7), while Alum adjuvant effects were independent of MyD88 (51).

LPD showed a good local safety due to the delivery of vaccines into skin MCs surrounded by normal skin. In our study, skin integrity could be recovered in 2 days and skin morphology could be recovered in 8 days following LPD (Supplemental Figure 5A). LPD also showed a good systemic safety. LPD of pdm09 vaccine failed to significantly increase body temperature of mice, while ID delivery significantly increased body temperature of mice (Supplemental Figure 6). The good systemic safety was likely due to its 12-hour sustained vaccine delivery (26). In further support, our unpublished data found LPD could minimize CpG adjuvant–induced cytokine storms. The good local and systemic safety support further development of LPD for more immunogenic vaccination.

Considering the AFL used in our study is an aesthetic device, AFL could be conveniently adapted for vaccine delivery in clinics. For mass immunization, a handheld AFL device could be fabricated and further integrate powder vaccine patch application. LPD is promising to significantly increase influenza vaccine efficacy in elderly people without increasing vaccine dose or incorporating chemical adjuvants. LPD is also promising to spare more influenza vaccine doses as compared with current ID delivery technologies. Besides influenza vaccines, LPD is promising to improve immunogenicity of other protein- or subunit-based vaccines that usually require vaccine adjuvants to induce protective immune responses. Besides improving vaccine immunogenicity, LPD may be able to minimize vaccine-induced systemic adverse reactions due to its sustained vaccine delivery. Direct powder vaccine delivery may eliminate vaccine reconstitution and improve vaccine stability. More immunogenic vaccination without the use of chemical adjuvants may relieve adjuvant safety concern and provide an alternative for those who prefer adjuvant-free vaccination. Our study supports the development of self-adjuvanted vaccine delivery technologies by intentional induction of well-controlled tissue stress to alert innate immune systems to boost vaccination.

## Methods

### Reagents.

Endotoxin-free OVA (EndoFit, <1 endotoxin unit/mg) and Alum adjuvant (Alhydrogel, 2%) were purchased from InvivoGen. AF647-OVA (O34784) was purchased from Thermo Fisher Scientific. The pdm09 vaccine (NR-20083) was obtained from Biodefense and Emerging Infections Research Resources Repository (BEI Resources). rHA of pdm09 strain (FR-559) was obtained from International Reagent Resource. DNase I (4536282001) was purchased from MilliporeSigma. Embryonic eggs (day 10, specific pathogen free) and chicken red blood cells (CRBCs) were purchased from Avian Vaccine Services from Charles River Laboratories.

### Animals and laser device.

BALB/c and C57BL/6 mice were purchased from Charles River Laboratories. DO11.10 (003303) and OT-II (004194) transgenic, NLRP3-KO (021302), and MyD88-KO mice (009088) were purchased from The Jackson Laboratory and self-bred for use in this study. Male mice at the age of 6–10 weeks old were used in this study. Animals were housed in animal facilities of University of Rhode Island (URI) and anesthetized for hair removal, laser treatment, and patch application. Lumenis UltraPulse ablative fractional CO_2_ laser was used to generate skin MCs with surrounding photothermal effects to boost vaccination.

### Powder OVA and pdm09 vaccine preparation.

Endotoxin-free OVA was mixed with mannitol at 1:25 ratio (w/w) and then lyophilized. To prepare pdm09 vaccine powder, pdm09 vaccine (0.5 mL) was concentrated to 0.125 mL by ultrafiltration with Amicon Ultra-0.5 Centrifugal Filter (3 kDa molecular weight cutoff). Half volume (~0.063 mL) of concentrated vaccine was mixed with 20% trehalose (0.125 mL) followed by lyophilization. Trehalose was used to maintain vaccine antigenicity during lyophilization and enhance vaccine delivery in LPD (26).

### Patch coating and extraction.

Adhesive patches of 750 μm thickness with a size of 6 × 6 mm^2^ were exposed to AFL to generate a 6 × 6 array of microcoating channels in 3 × 3 mm^2^ as in our previous report (26). Vaccine powder was pushed into microcoating channels until full. Patch-coated vaccines were extracted into phosphate-buffered saline (PBS) for use in ID delivery. OVA coating amount was measured by BCA protein assay kit (23227, Thermo Fisher Scientific). To quantify pdm09 vaccine coating amount, extracted pdm09 vaccine and standard pdm09 vaccine with known hemagglutinin antigen (HA) contents were subjected to SDS-PAGE. Band intensity of HA was used to quantify pdm09 vaccine amount and found to be approximately 0.15 μg HA per patch.

### Immunization.

Hair on the lateral dorsal skin of mice was removed 1 day before the experiment as in our previous report (52). For LPD, hair-removed skin was treated with AFL to generate a 6 × 6 array of skin MCs in 3 × 3 mm^2^ followed by topical application of powder OVA or pdm09 vaccine–coated array patches. Multiple AFL treatments and multiple patches could be applied to increase vaccine dose when needed. Patches were firmly pressed on the skin to ensure a good delivery. A narrow bandage was used to keep patches in position for 2 days. For ID delivery, patch extracts were intradermally injected into AFL- or sham-treated skin to ensure the delivery of the same antigen amount. ID delivery was administered with insulin syringes equipped with 29G needles. In sham treatment, mice underwent the same procedures except the laser was not activated. A 3M Tegaderm film was applied prior to ID vaccine injection to prevent leakage.

### Immune cell recruitment.

Lateral back skin of BALB/c mice was exposed to AFL treatment to generate a 12 × 12 array of skin MCs in 6 × 6 mm^2^ or subjected to ID injection of 20 μL Alum adjuvant (160 μg aluminum contents), which occupied a circular area of approximately 6 mm in diameter. AFL-treated and Alum-injected skin of 6 × 6 mm^2^ was dissected 6, 12, 24, 48, 72, and 120 hours later followed by single-cell suspension preparation; immunostaining with fluorescence-conjugated antibodies against Ly6C (clone HK1.4), CD11b (clone M1/70), CD11c (clone N418), MHC II (clone M5/114.15.2), Ly6G (clone 1A8), and F4/80 (clone BM8), all from BioLegend; and flow cytometry analysis of percentage of neutrophils (CD11b^+^Ly6G^hi^Ly6C^+^F4/80^–^), monocytes (CD11b^+^Ly6G^–^Ly6C^+^F4/80^int^), macrophages (CD11b^+^F4/80^hi^), eosinophils (CD11b^+^Ly6G^int^Ly6C^−^F4/80^int^), and mDCs (CD11b^+^CD11c^+^MHCII^hi^F4/80^lo^) as in our recent report (44).

### Antibody titer measurement.

Serum antibody titer was measured by enzyme-linked immunosorbent assay (ELISA). In brief, OVA (100 μg/mL) was coated into 96-well ELISA plates at 4°C overnight. After blocking with 5% nonfat milk, 2-fold serial dilutions of immune sera were added and incubated at room temperature for 90 minutes. After washing in PBS supplemented with 0.05% Tween 20 (PBST), HRP-conjugated anti-mouse IgG (NA931, GE Healthcare Life Sciences, now Cytiva) or subtype IgG1 (046120, Invitrogen, Thermo Fisher Scientific), IgG2a (NB7516, Novus Biologicals), or IgG2c antibodies (A90-136P, Bethyl Laboratories) were added and incubated at room temperature for 1 hour. After washing in PBST, 3,3′,5,5′-tetramethylbenzidine substrates were added, and reactions were then stopped by addition of 3 M H_2_SO_4_. Optical absorbance (OD_450/650 nm_) was read in a microplate reader (Molecular Devices). Serum antibody titer was defined as the reciprocal dilution factor that resulted in OD_450/650 nm_ that was approximately 3 times higher than the background values.

### HI titer.

Influenza virus propagation, hemagglutinating unit determination, and measurement of HI titer were performed with reference to our previous report (44). To measure HI titer, serum samples were incubated with receptor-destroying enzyme followed by heat inactivation to remove complement activity. Serum samples were further adsorbed with CRBCs to remove nonspecific binding. The resultant serum samples were subjected to a 2-fold serial dilution and incubated with 4 hemagglutinating units of pdm09 virus (A/California/07/2009) and then 1% CRBCs. HI titer was defined as the reciprocal of the highest dilution that completely inhibited agglutination of CRBCs.

### Influenza challenge.

Influenza viral challenges referred to our previous report (44). Briefly, mice were anesthetized and inoculated intranasally with 10 × LD_50_ of mouse-adapted influenza viruses. Body weight and survival were monitored daily for 14 days. Mice were euthanized and considered to have died if their body weight loss was more than 25%.

### Cytokine array assay.

Cytokine levels in skin homogenates were measured with the mouse Cytokine Array Panel A (ARY006, R&D Systems, Bio-Techne). In brief, BALB/c mice were subjected to AFL or ID injection of Alum adjuvant (160 μg aluminum content) or left nontreated. Skin was dissected 24 hours later and homogenized in T-PER Reagent (78510, Thermo Fisher Scientific) followed by centrifugation at 18,000*g* for 10 minutes at 4°C. Supernatants were pooled for analysis of cytokine levels. In brief, membranes coated in duplicate with capturing antibodies against 40 cytokines were blocked and then incubated with tissue homogenates in the presence of a cocktail of biotinylated detection antibodies. After washing, membranes were incubated with HRP-conjugated antibodies (NA931, GE Healthcare Life Sciences, now Cytiva). After washing, membranes were incubated with SuperSignal West Femto Maximum Sensitivity Substrate (34095, Thermo Fisher Scientific) and then imaged under myECL Imager (Thermo Fisher Scientific).

### Western blotting.

Proteins were separated in SDS-PAGE and then transferred to a PVDF membrane followed by blocking with 5% nonfat milk. The PVDF membrane was incubated with goat anti-mouse IL-1β antibody (AF-401-NA, R&D Systems, Bio-Techne). After washing, the PVDF membrane was incubated with HRP-conjugated anti-goat antibody (HAF017, R&D Systems, Bio-Techne). After washing, the PVDF membrane was incubated with SuperSignal West Femto Maximum Sensitivity Substrate (Thermo Fisher Scientific) and imaged under myECL Imager (Thermo Fisher Scientific). The PVDF membrane was then stripped in stripping buffer (62 mM Tris-HCl at pH 6.8, 2% SDS, 100 mM β-mercaptoethanol) for other protein detection. In brief, the stripped PVDF membrane was blocked, incubated with rabbit anti-GAPDH antibodies (5174s, Cell Signaling Technology) or rabbit anti–Caspase-1 antibodies (AB1871, MilliporeSigma), followed by the same procedures for detection of GAPDH and Caspase-1, except HRP-conjugated anti-rabbit antibodies (7074P2, Cell Signaling Technology) were used as secondary antibodies.

### Single-cell preparation, staining, and flow cytometry.

Skin single-cell suspensions were prepared by subjecting skin to collagenase D (0.2%) and dispase (0.6 U/mL) digestion as in our previous report (44). LNs were passed through 40 μm cell strainers to prepare single-cell suspensions. For DC subset analysis, skin cells were first stained with fixable viability dye eFluor 450 (eBioscience, Thermo Fisher Scientific) except as otherwise specified. Skin and LN cells were then stained with fluorescence-conjugated anti-CD11c (clone N418), MHC II (clone M5/114.15.2), Langerin (clone 4C7), CD11b (clone M1/70), CD103 (clone 2E7), and CD80 antibodies (clone 16-10A1), all from BioLegend. For intracellular cytokine staining, LN cells were stimulated with 10 μg/mL OVA or 1 μg/mL OVA_323-339_ (vac-isq, InvivoGen) in the presence of anti-CD28 antibodies (clone 37.51, BioLegend) overnight. Brefeldin A (420601, BioLegend) was added 5 hours before cell harvest. Cells were stained with fluorescence-conjugated anti-CD4 (clone GK1.5) and anti-CD8 antibodies (clone 53-6.7) or fluorescence-conjugated anti-mouse TCR DO11.10 (KJ1-26) and anti-CD4 antibodies (clone GK1.5), fixed and permeabilized, and further stained with fluorescence-conjugated anti–IL-4 (clone 11B11) and anti–IFN-γ antibodies (clone XMG1.2). (All antibodies were obtained from BioLegend.) LN cells in the adoptive DO11.10 T cell transfer experiment were also directly stained with fluorescence-conjugated KJ1-26, anti-CD4, and anti-CD8 antibodies. Cells were subjected to flow cytometry analysis in BD FACSVerse.

### Adoptive transfer.

LNs and spleens were harvested from DO11.10 or OT-II transgenic mice, in which CD4^+^ T cells recognize OVA residues 323–339 in the context of H2^d^ and I-A^b^, respectively. LNs and spleen were passed through 40 μm cell strainers to prepare single-cell suspensions. After red blood cell lysis, naive CD4^+^ T cells were purified by subjecting cells to magnetic bead–based negative purification with a commercial kit (130-104-453, Miltenyi Biotec). CD4^+^ T cells were then stained with 5 μM CFSE (C34554, Thermo Fisher Scientific), thoroughly washed in PBS, and adjusted to 10^7^ cells/mL in PBS. A total of 10^6^ CFSE-labeled transgenic CD4^+^ T cells were intravenously injected into host BALB/c or C57BL/6 mice.

### Histological and in situ apoptosis analysis.

AFL-treated skin was dissected, fixed in formalin, and subjected to paraffin embedding and sectioning. Paraffin sections were subjected to side-by-side H&E staining and in situ apoptosis analysis via a commercial kit (ab206386, Abcam). In brief, paraffin sections were deparaffinized in xylene and rehydrated in a graded alcohol series. After being treated with proteinase K, the sections were incubated with 3% H_2_O_2_ to inactivate endogenous peroxidases. Biotin labeling of exposed 3′-OH ends of DNA of apoptotic cells was generated by adding terminal deoxynucleotidyl transferase (TdT) enzyme and TdT labeling reaction mix (included in the kit) for 90 minutes at room temperature, and the reaction was stopped using the stop buffer. Biotinylated DNA was detected by incubation with a streptavidin-HRP conjugate (included in the kit) for 30 minutes at room temperature. After washing in TBS, DAB substrate was added, and reactions were stopped. The sections were counterstained with Methyl Green, dehydrated, and then coverslipped. Images were taken under a Nikon Eclipse E600 microscope.

### Double-stranded DNA detection.

For double-stranded DNA detection, 100 μL of DRAQ7 (10 μM, BD Pharmingen) was subcutaneously injected into AFL-treated skin following a published protocol (39). Skin was dissected 30 minutes later and subjected to cryosectioning. Cryosections were imaged under a Nikon Eclipse Ti2 inverted confocal microscope.

### Real-time PCR.

Total RNA was isolated with TRIzol method and reverse-transcribed as shown in our previous report (44). Relative gene expression of cytokines and chemokines was analyzed by real-time PCR in Applied Biosystems ViiA 7 (Thermo Fisher Scientific) using GAPDH as internal control. Primer sequences of each gene are listed in Supplemental Table 1.

### Statistics.

Values were expressed as mean ± SEM. Student’s *t* test or Mann-Whitney *U* test was used to analyze differences between groups. One-way ANOVA with Tukey’s or Newman-Keuls multiple-comparison test unless otherwise specified was used to compare differences among groups. Two-way ANOVA with Bonferroni’s posttest was used to compare differences between groups at different time points or as otherwise specified. Log-rank (Mantel-Cox) test with Bonferroni’s correction was used to compare survival difference between groups. *P* value was calculated by Prism software (GraphPad) and considered significant if it was less than 0.05.

### Study approval.

All animal procedures were approved by the Institutional Animal Care and Use Committee of URI with protocol number AN#1415-009.

## Author contributions

XC designed experiments; ZL, YC, YL, and YZ conducted experiments and acquired data; ZL, YC, and XC analyzed data; and XC wrote the manuscript. The order of co–first authors reflects the relative time contributed by each author.

## Supplementary Material

Supplemental data

## Version 1 03/09/2021

In-Press Preview

## Version 2 04/08/2021

Electronic publication
