# Supplementary material for: Vaccine delivery alerts innate immune systems for more immunogenic vaccination
Source: JCI Insight. 2021 Apr 8;6(7):e144627. doi: 10.1172/jci.insight.144627 (PMC8119203; doi:10.1172/jci.insight.144627)
Supplement: Supplemental data [file jciinsight-6-144627-s108.pdf]

Supplementary Information

Vaccine delivery alerts innate immune systems for more immunogenic vaccination

Li et. al.

Supplementary Materials

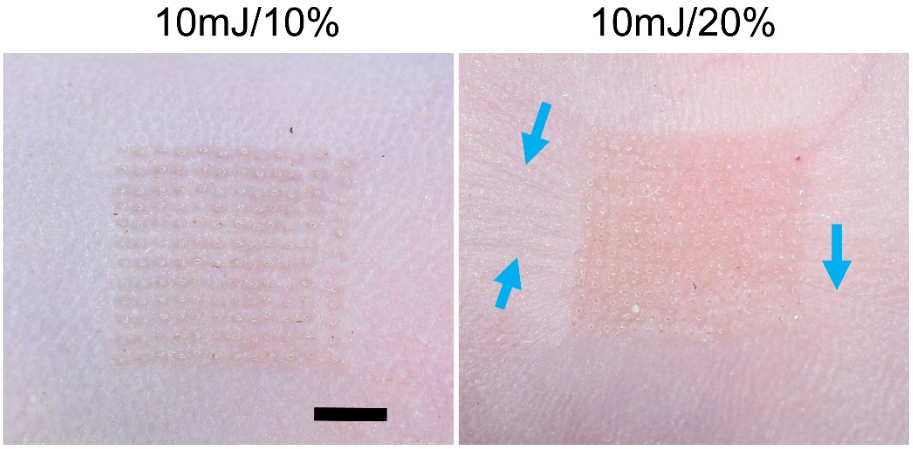

Suppl. Fig.1 AFL (10mJ/20%) induces skin shrinkage
Lateral back skin of BALB/c mice (6 × 6 mm^2) was exposed to AFL at 10 mJ energy and 10 or 20% coverage. Skin pictures were taken right after AFL treatment. Representative skin pictures of 3 repeats were shown. Arrows point to signs of skin shrinkage. Scale: 2 mm.

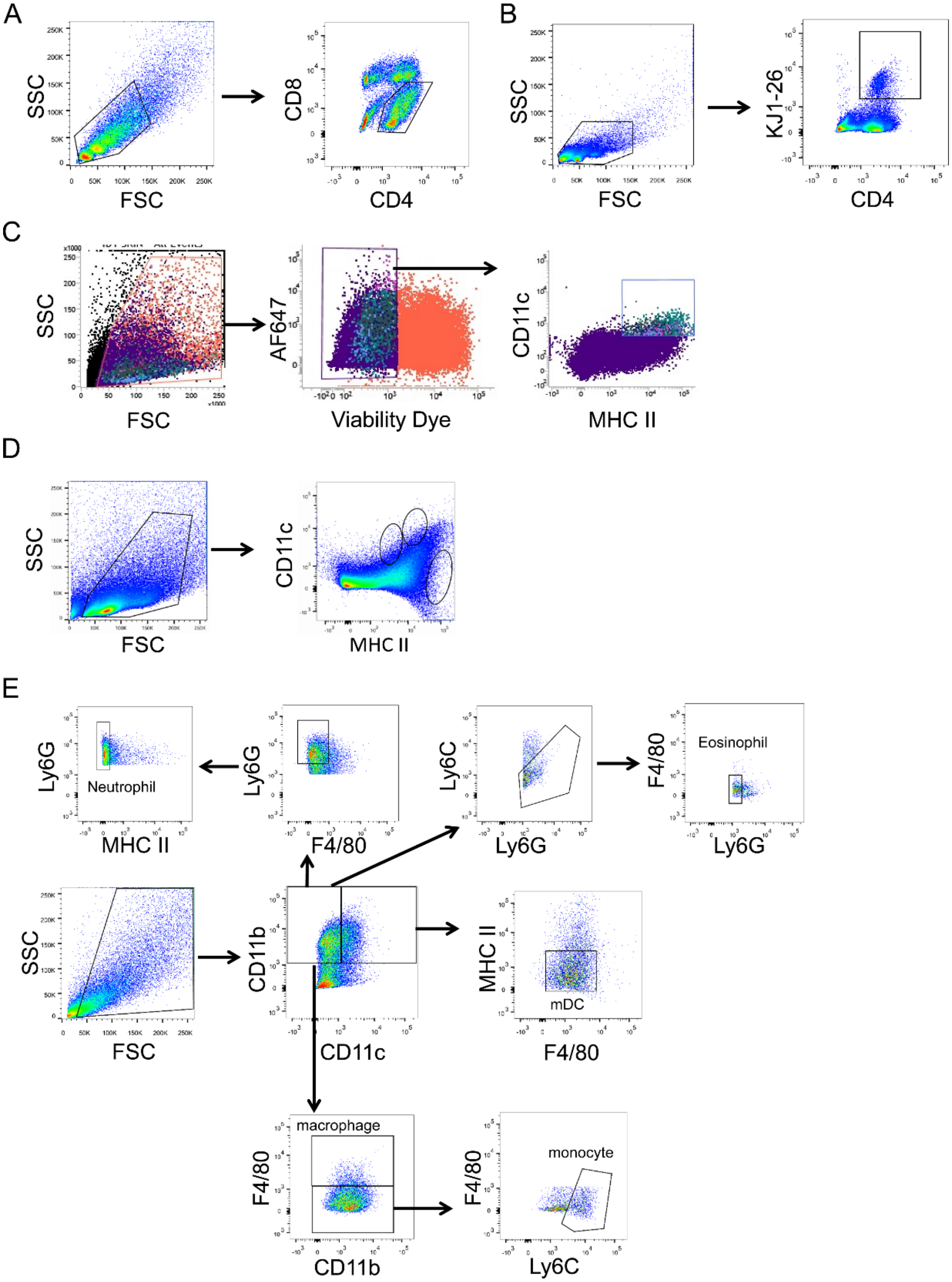

Suppl. Fig. 2 Gating strategies in flow cytometry analysis

A. Gating strategy to identify $CD4^+$ T cells in Fig.1D. **B**. Gating strategy to identify KJ1-26^+ cells in Fig.1F. **C**. Gating strategy to identify skin DC subsets in Fig.3A and 8A. **D**. Gating strategy to identify DC subsets in draining LNs in Fig.4A and 8B. **E**. Gating strategy to identify innate immune cells in Fig.5E.

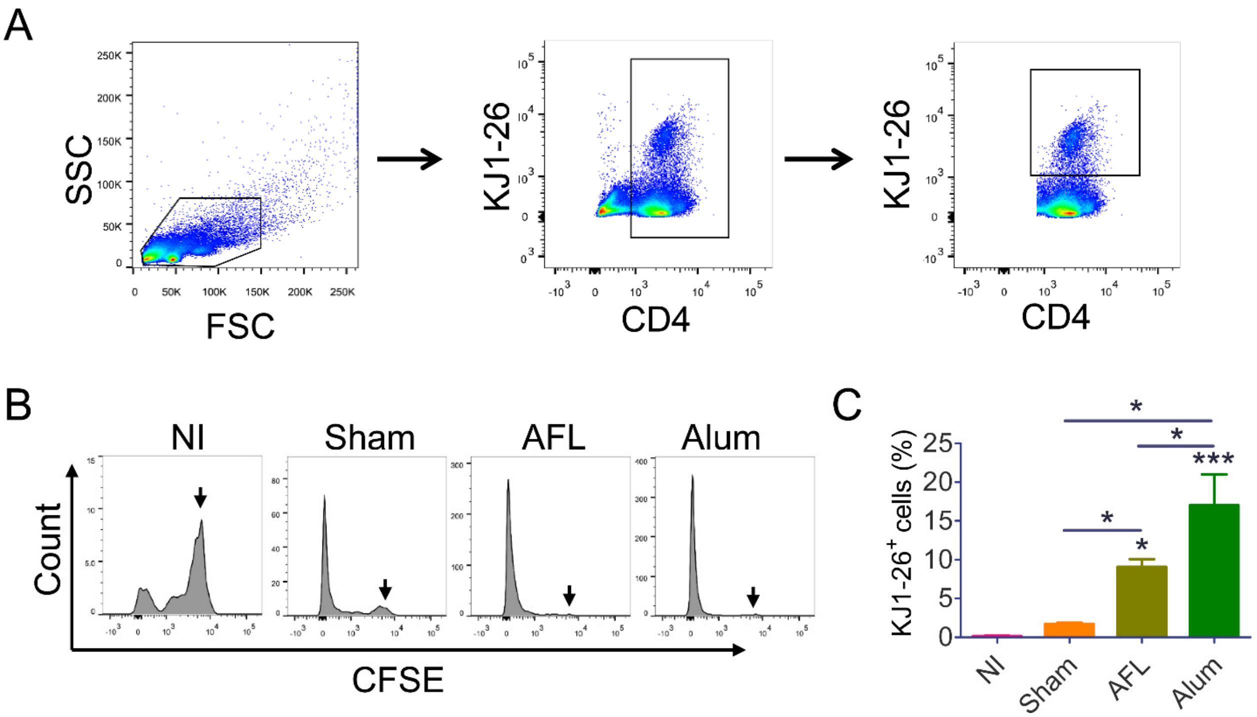

Suppl. Fig. 3 Division of adoptively transferred CFSE-labeled transgenic CD4$^+$ T cells from DO11.10 mice

A. Gating strategy to evaluate percentage of KJ1-26$^+$ cells in CD4$^+$ T cells. Experimental procedures were described in Fig.1D. **B**. Representative histogram to indicate division of CFSE-labeled transgenic CD4$^+$ T cells in different groups. Arrows point to non-divided CD4$^+$ T cells. **C**. Percentage of KJ1-26$^+$ cells in CD4$^+$ T cells. One-way ANOVA with Newman-Keuls multiple comparison test was used to compare differences between groups. n=4. *, $p<0.05$; ***, $p<0.001$.

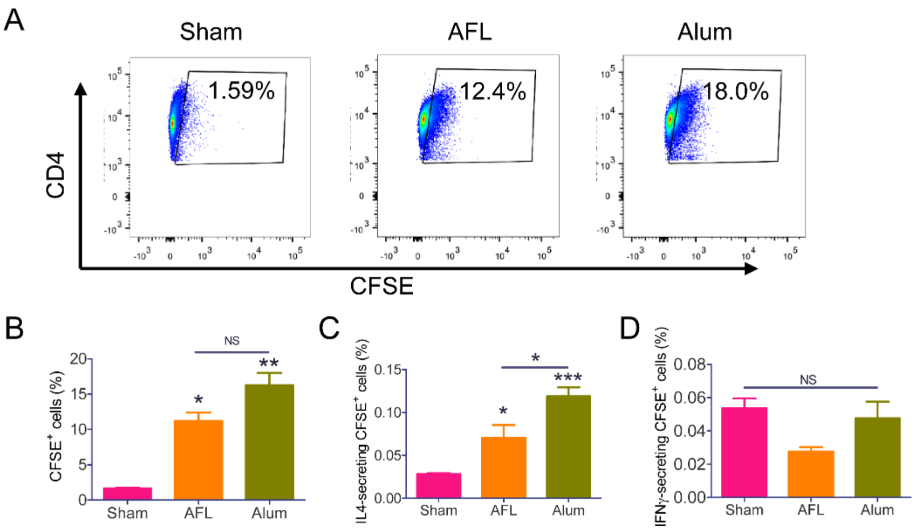

Suppl. Fig. 4 Expansion of adoptively transferred OT II T cells following in vitro stimulation

CFSE-stained OT II cells were adoptively transferred to C57BL/6 mice followed by ID injection of 10 µg OVA with or without prior AFL treatment or in the presence of Alum adjuvant. LN cells were harvested 4 days later and stimulated with $OVA_{323-339}$ peptide followed by intracellular cytokine staining and flow cytometry analysis. **A**. Representative dot plots showing percentage of $CFSE^+$ cells in $CD4^+$ T cells. **B**. Percentage of $CFSE^+$ cells in $CD4^+$ T cells in different groups. **C-D**. Percentage of IL4 and IFNγ-secreting $CFSE^+$ cells in $CD4^+$ T cells was shown in **C** and **D**, respectively. One-way ANOVA with Tukey's multiple comparison test was used to compare differences between groups in **B-D**. n=4. *, p<0.05; **, p<0.01; ***, p<0.001. NS: not significant.

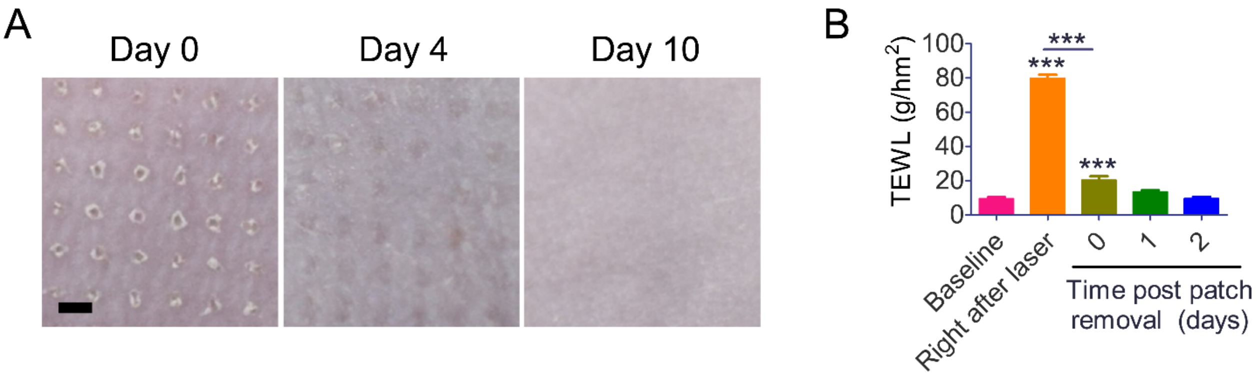

Suppl. Fig. 5 Local reactions following LPD of pdm09 vaccine

BALB/c mice were subjected to AFL treatment followed by topical application of powder pdm09 vaccine-coated patches. Patches were removed 2 days later. **A**. Representative skin pictures right after and 4 and 10 days after AFL treatment. Scale: 600 µm. **B**. TEWL value of the skin before and right after AFL treatment and right after and 1 and 2 days after patch removal. n=5-8. One-way ANOVA with Newman-Keuls multiple comparison test was used to compare differences between groups in **B**. ***, $p<0.001$.

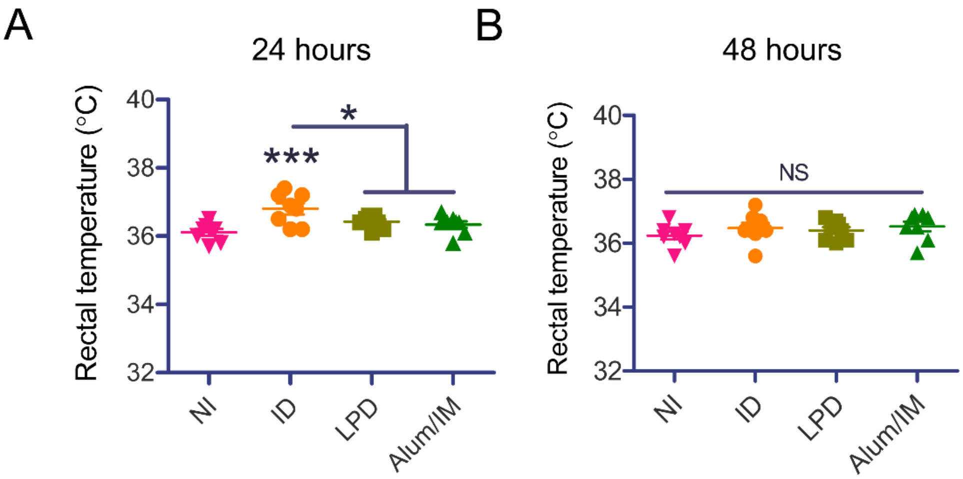

Suppl. Fig. 6 Rectal temperature following the different immunizations
C57BL/6 mice were subjected to LPD or ID delivery of pdm09 vaccine, or IM delivery of pdm09 vaccine in the presence of Alum adjuvant (Alum/IM), or left non-immunized (NI). Rectal temperature of mice was measured 24 (**A**) and 48 hours (**B**) after immunization. n=8. One-way ANOVA with Newman-Keuls multiple comparison test was used to compare differences between groups. *, $p<0.05$; ***, $p<0.001$. NS: not significant.

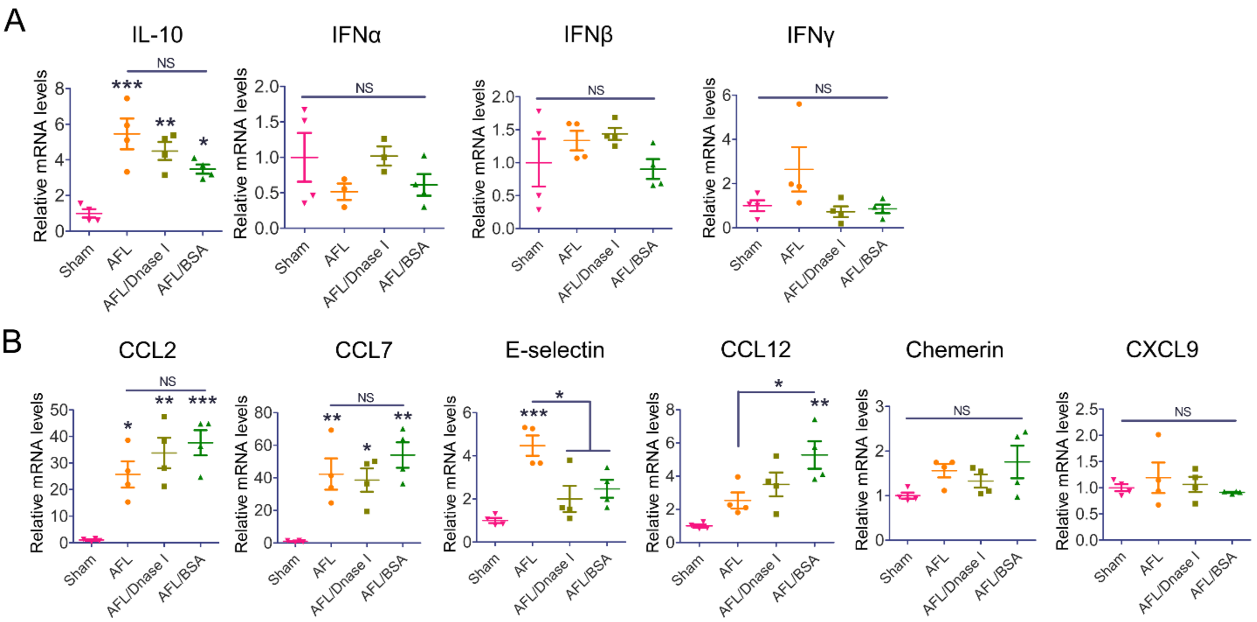

Suppl. Fig. 7 Relative mRNA levels of different cytokines and chemokines

C57BL/6 mice were subjected to AFL or sham treatment or AFL treatment following by ID injection of DNase I or BSA into AFL-treated skin. Skin was dissected 6 hours later and subjected to real-time PCR analysis of the relative mRNA levels of different cytokines (**A**) and chemokines (**B**). n=4. One-way ANOVA with Newman-Keuls multiple comparison test was used to compare differences between groups. *, p<0.05; **, p<0.01; ***, p<0.001. NS: not significant.

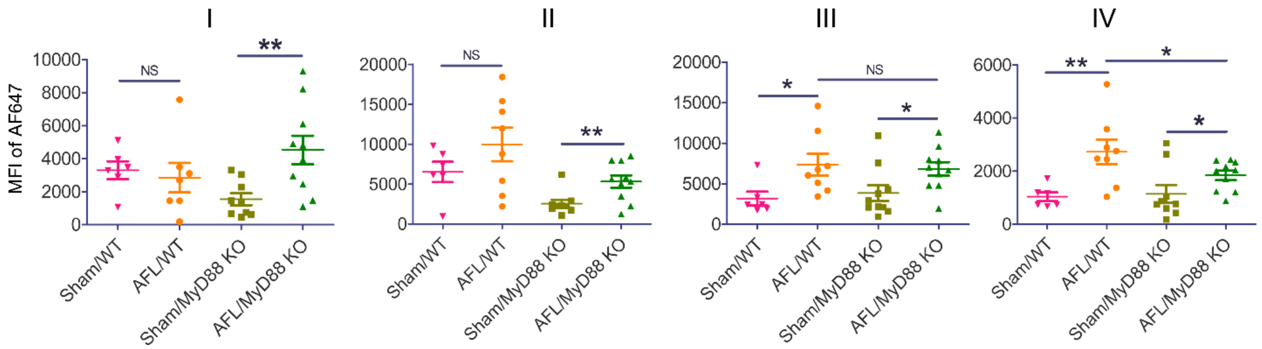

Suppl. Fig. 8 Antigen uptake and maturation of skin DC subsets

WT and MyD88 KO mice were subjected to AFL or sham treatment followed by ID injection of AF647-OVA into AFL or Sham-treated skin. Antigen uptake was analyzed in individual skin DC subsets as in Fig.8A. MFI of AF647 in different skin DC subsets was shown. One-tailed student t-test was used to compare differences between groups. n=6-10. *, $p<0.05$; **, $p<0.01$. NS: not significant.

Suppl. Table 1. Primer sequences used in real-time PCR (5’-3’)

Gene	Forward	Reverse
CCL2	TTAAAAACCTGGATCGGAACCAA	GCATTAGCTTCAGATTTACGGGT
CCL7	GATCTCTGCCACGCTTCTGT	ATAGCCTCCTCGACCCACTT
CCL12	GTCCTCAGGTATTGGCTGGA	CACTGGCTGCTTGTGATTCT
CXCL9	GGAGTTCGAGGAACCCTAGTG	GGGATTTGTAGTGGATCGTGC
CHEMERIN	GTGCACAATCAAACCAAACG	GGCAAACTGTCCAGGTAGGA
E-SELECTIN	ATGCCTCGCGCTTTCTCTC	GTAGTCCCGCTGACAGTATGC
IL-1α	CGAAGACTACAGTTCTGCCATT	GACGTTTCAGAGGTTCTCAGAG
IL-1β	GCAACTGTTCCTGAACTCAACT	ATCTTTTGGGGTCCGTCAACT
IL-6	TAGTCCTTCCTACCCCAATTTCC	TTGGTCCTTAGCCACTCCTTC
IL-10	GCTCTTACTGACTGGCATGAG	CGCAGCTCTAGGAGCATGTG
IFNA4 (IFNα)	TGATGAGCTACTACTGGTCAGC	GATCTCTTAGCACAAGGATGGC
IFNβ	CAGCTCCAAGAAAGGACGAAC	GGCAGTGTAACTCTTCTGCAT
IFNγ	ATGAACGCTACACACTGCATC	CCATCCTTTTGCCAGTTCCTC
TNFα	CCCTCACACTCAGATCATCTTCT	GCTACGACGTGGGCTACAG
GAPDH	AGGTCGGTGTGAACGGATTTG	TGTAGACCATGTAGTTGAGGTCA

Suppl. Table 2. Statistical analysis of body weight difference in Fig.2K

	ID	Alum/IM	LPD
NI	** (Day 5-6)	*** (Day 4-6)	* (Day 3); *** (Day 4-6)
ID	-	NS	*** (Day 6-7); ** (Day 8)
Alum/IM	-	-	*** (Day 7)

(Note: Two-way ANOVA with Bonferroni post-test was used to compare body weight difference between groups. *, $p<0.05$; **, $p<0.01$; ***, $p<0.001$. NS: not significant)
